# Single-cell and bulk RNAseq unveils the immune infiltration landscape and targeted therapeutic biomarkers of psoriasis

**DOI:** 10.3389/fgene.2024.1365273

**Published:** 2024-04-18

**Authors:** Wenhui Deng, Yijiao Yan, Chengzhi Shi, Daoshun Sui

**Affiliations:** ^1^ The First Clinical Medical College, Guangzhou University of Chinese Medicine, Guangzhou, Guangdong, China; ^2^ The First Affiliated Hospital of Chinese Medicine, Guangzhou University of Chinese Medicine, Guangzhou, Guangdong, China

**Keywords:** psoriasis, biomarker, immune, scRNAseq, machine learning, molecular subtype

## Abstract

**Background:**

Psoriasis represents a multifaceted and debilitating immune-mediated systemic ailment afflicting millions globally. Despite the continuous discovery of biomarkers associated with psoriasis, identifying lysosomal biomarkers, pivotal as cellular metabolic hubs, remains elusive.

**Methods:**

We employed a combination of differential expression analysis and weighted gene co-expression network analysis (WGCNA) to initially identify lysosomal genes. Subsequently, to mitigate overfitting and eliminate collinear genes, we applied 12 machine learning algorithms to screen robust lysosomal genes. These genes underwent further refinement through random forest (RF) and Lasso algorithms to ascertain the final hub lysosomal genes. To assess their predictive efficacy, we conducted receiver operating characteristic (ROC) analysis and verified the expression of diagnostic biomarkers at both bulk and single-cell levels. Furthermore, we utilized single-sample gene set enrichment analysis (ssGSEA), CIBERSORT, and Pearson’s correlation analysis to elucidate the association between immune phenotypes and hub lysosomal genes in psoriatic samples. Finally, employing the Cellchat algorithm, we explored potential mechanisms underlying the participation of these hub lysosomal genes in cell-cell communication.

**Results:**

Functional enrichment analyses revealed a close association between psoriasis and lysosomal functions. Subsequent intersection analysis identified 19 key lysosomal genes, derived from DEGs, phenotypic genes of WGCNA, and lysosomal gene sets. Following the exclusion of collinear genes, we identified 11 robust genes, further refined through RF and Lasso, yielding 3 hub lysosomal genes (S100A7, SERPINB13, and PLBD1) closely linked to disease occurrence, with high predictive capability for disease diagnosis. Concurrently, we validated their relative expression in separate bulk datasets and single-cell datasets. A nomogram based on these hub genes may offer clinical advantages for patients. Notably, these three hub genes facilitated patient classification into two subtypes, namely metabolic-immune subtype 1 and signaling subtype 2. CMap analysis suggested butein and arachidonic fasudil as preferred treatment agents for subtype 1 and subtype 2, respectively. Finally, through Cellchat and correlation analysis, we identified PRSS3-F2R as potentially promoting the expression of hub genes in the psoriasis group, thereby enhancing keratinocyte-fibroblast interaction, ultimately driving psoriasis occurrence and progression.

**Conclusion:**

Our study identifies S100A7, SERPINB13, and PLBD1 as potential diagnostic biomarkers, offering promising prospects for more precisely tailored psoriatic immunotherapy designs.

## Introduction

Psoriasis is a prevalent autoimmune inflammatory disorder that impacts millions of people worldwide, with a prevalence rate of 2%–3% (Damiani et al., 2021; Branisteanu et al., 2022). The diagnosis of psoriasis is based on the characteristic well-demarcated, erythematous, and pruritic plaques covered with silvery scales (Rendon and Schakel, 2019). However, the impact of psoriasis extends beyond the skin, with associated manifestations in nails and joints, among others. The release of pro-inflammatory cytokines into systemic circulation is responsible for a range of comorbidities, including metabolic syndrome (MetS), cardiovascular disease (CVD), inflammatory bowel disease (IBD), and malignancy, among others (Tashiro and Sawada, 2022; Wu et al., 2022). Psoriasis is a chronic systemic disease that negatively affects patients’ quality of life, leading to a significant disease burden. The understanding of the systemic effects of psoriasis and the identification of comorbidities are crucial to improving patient outcomes and reducing the disease burden.

Psoriasis is a multifaceted disorder that results from the intricate interplay of multiple factors, including immune dysregulation, host genetics, environmental triggers, and skin barrier disruption (Bochenska and Gabig-Ciminska, 2020). Despite extensive research, the precise mechanisms underlying psoriasis pathogenesis remain unclear. However, it is well-established that the sustained inflammation, hyperproliferation, and abnormal differentiation of epidermal keratinocytes are key hallmarks of the disease. In particular, T cells and their cytokines, such as TNF-α, IL-23, IL-12, and IL-6, are known to play a central role in psoriasis pathogenesis, leading to the activation of cascades of inflammatory responses that promote keratinocyte proliferation and neutrophil recruitment (Jadali and Eslami, 2014; Gran et al., 2020). Recent research has highlighted the critical role of autophagy pathways in regulating inflammatory responses in psoriasis (Reveille, 2011; Yin et al., 2018). Autophagy, a collection of lysosomal processes that contribute to intracellular homeostasis, has emerged as a potential and promising therapeutic target for the treatment of psoriasis (Deretic, 2021; Yang and Wang, 2021). In particular, Lee et al., 2011 demonstrated that blockade of autophagy process promoted the relative expression of p62 and generation of inflammatory cytokines in primary human keratinocytes. Moreover, a recent study has confirmed that prolonged exposure to the pro-inflammatory cytokine TNF-α decreases the levels of major cathepsins in lysosomes, leading to impaired autophagy (Klapan et al., 2022). Therefore, lysosomes and autophagy pathways represent critical targets for therapeutic intervention in psoriasis.

In recent years, there has been a burgeoning interest in elucidating the contribution of lysosomes to autoimmune pathologies (Ge et al., 2015; Kimura et al., 2017). Among cellular organelles, lysosomes emerge as pivotal entities implicated in orchestrating the inflammatory cascade. The seminal link between lysosomes and cutaneous inflammation was first delineated through seminal investigations during the 1970s (Chayen and Bitensky, 1971; Winkelmann, 1971; Lazarus et al., 1975). Subsequent studies have yielded pivotal insights into the intricate involvement of lysosomes in modulating inflammatory responses and autoimmune disorders (He et al., 2011; Reinheckel, 2013; Ballabio, 2016), thereby elucidating a lysosome-to-nucleus signaling axis and a regulatory network of lysosomal genes governing cellular clearance and metabolic homeostasis. Furthermore, mounting evidence underscores the disruptive impact of aberrant lysosomal function on immune dysregulation and inflammatory manifestations (Ge et al., 2015; Sa and Festa, 2016). For instance, He et al., 2011 uncovered the pivotal role of lysosomes in modulating glucocorticoid signaling pathways, thereby laying a mechanistic foundation for combinatorial therapeutic approaches utilizing glucocorticoids and lysosomal inhibitors to ameliorate inflammation and autoimmune maladies. Nevertheless, the quest for discerning lysosomal-associated biomarkers in psoriasis remains incomplete, underscoring a pressing need for further investigation. Similarly, our comprehension of the intricate mechanisms underpinning the involvement of lysosomal genes in mediating cell-cell interactions is still in its incipient stage, warranting continued exploration and scrutiny.

Bioinformatic approaches are becoming increasingly important in unraveling the complex mechanisms underlying various diseases (Zhou et al., 2020; Wu Z. et al., 2021; Liang et al., 2021). In the context of psoriasis, Li et al., 2023 conducted comprehensive bioinformatic analyses, culminating in the identification of five putative hub genes (SOD2, PGD, PPIF, GYS1, and AHCY) associated with psoriasis, as confirmed through RT-qPCR and immunohistochemistry. Similarly, Yue et al., 2022 elucidated several biomarkers with therapeutic potential in psoriasis. Moreover, IFIT3 emerged as a novel regulatory factor implicated in psoriasis pathogenesis (Li et al., 2022).

In our study, we employed multiple bioinformatic methods to identify promising molecular lysosomal biomarkers, and seek potential molecular targets for precision therapy in psoriasis. By focusing on vital lysosomal genes and searching for diagnostic targets, the study sheds light on the correlation of diagnostic targets with immune cells and immune responses, thus providing new insights into the pathogenesis of psoriasis. These findings highlight the crucial role of lysosomes in regulating immune microenvironment of psoriasis. In conclusion, a deeper understanding of the heterogeneity of the immune microenvironment in psoriasis and the role of lysosomes in regulating autophagy may provide new insights into the development and treatment of psoriasis.

## Materials and methods

### Data sources and processing

**FIGURE 1:**
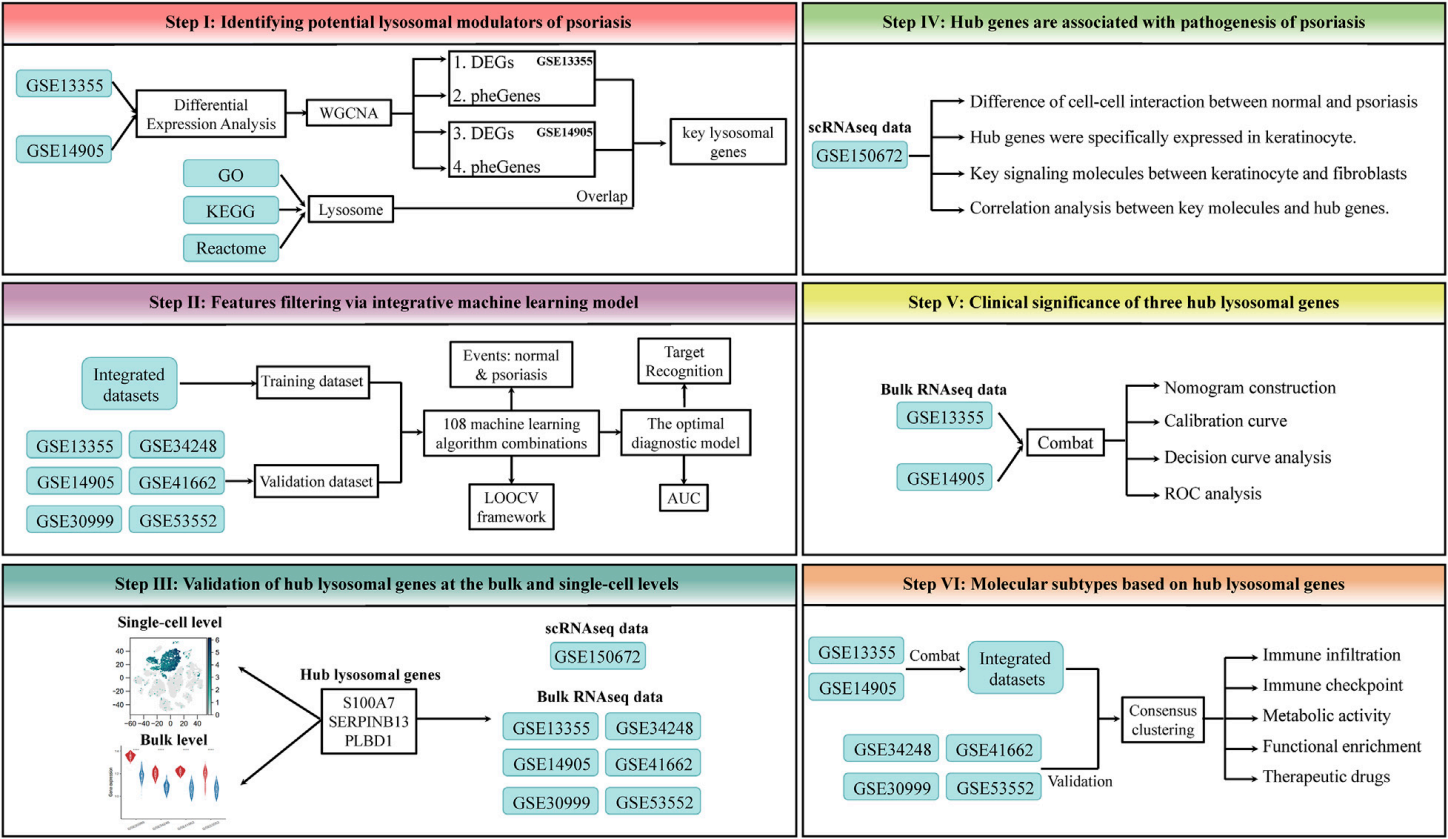
The workflow of this study. This framework mainly includes the following steps: 1) Identifying potential lysosomal modulators of psoriasis. 2) Features filtering via integrative machine learning model. 3) Validation of hub lysosomal genes at the bulk and single-cell levels. 4) Hub genes are associated with pathogenesis of psoriasis. 5) Clinical significance of three hub lysosomal genes. 6) Molecular subtypes based on hub lysosomal genes.

In this study, the expression spectrums of bulk RNAseq data derived from Lesion Skin (LS), Uninvolved Skin (US), and Normal Skin (NS) samples were obtained from six public datasets, GSE13355 (64 NS, 58 US and 58 LS samples) (Nair et al., 2009) and GSE14905 (21 NS, 28 US and 33 LS samples) (Yao et al., 2008), GSE30999 (85 US and 85 LS samples) (Suarez-Farinas et al., 2012), GSE34248 (14 US and 14 LS samples) (Bigler et al., 2013), GSE41662 (24 US and 24 LS samples) (Bigler et al., 2013), and GSE53552 (24 US and 75 LS samples) (Russell et al., 2014). The corresponding annotation file was utilized to convert the probe ID to the gene symbol. If a gene possesses multiple corresponding probes, the average value of these probes will be calculated. Furthermore, those probes without matching genes were removed from the analysis to ensure accurate results. Subsequently, a set of 866 lysosomal genes was identified from the Gene Ontology (GO), Kyoto Encyclopedia of Genes and Genomes (KEGG), and Reactome database (Supplementary Table S1). Due to the detailed phenotypic information provided by GSE13355 and GSE14905, they were used to identify the initial key lysosomal genes. Notably, a total of datasets of this study was integrated through *Combat* function of the “sva” package, and the integrated dataset was used as the training set for the classification model, and each individual dataset was used as the validation set. The detailed workflow of this study was shown in Figure 1.

### Differential expression analysis and functional enrichment analysis

Considering the potential batch effect from different datasets, we adopted a strategy of conducting differential expression analysis on individual datasets and then taking intersections of differentially expressed genes (DEGs). The “limma” (Ritchie et al., 2015) package was employed to identify DEGs between LS and NS samples obtained from GSE13355 and GSE14905, and the significant threshold was set as FDR value <0.05 and |log2 fold change (FC)| > 1, ensuring robust and reliable results. To visualize the DEGs, we utilized the “ggplot2” packages to generate visually volcano plots. Next, we narrowed down the DEGs to those that overlapped between the two datasets, allowing us to identify the most significant genes related to the differences between LS and NS. Then, the overlap DEGs were utilized for functional enrichment analysis (GO and KEGG) to determine the major biological terms through “clusterProfiler” (Wu T. et al., 2021) package. The enrichment results were further visualized using “ggplot2” and “ggraph” packages.

## Evaluating immune cell infiltrations

Based on gene expression profiles of the integrated dataset, we employed the “CIBERSORT” (Newman et al., 2015) approach to identify relative immune cell composition in tissues. Only samples with *p*-values less than 0.05 were regarded as accurate immune cell fraction analysis. Additionally, we utilized correlation analysis to examine the relevance between three hub genes and immune cell infiltration. To infer the relative abundance of immune-related and hallmark gene sets between different groups, we utilized the single-sample gene set enrichment analysis (ssGSEA) algorithm. The Wilcoxon test was utilized for evaluating the differences in gene sets or immune cell proportions between NS and US or LS. One asterisk (*) represents *p*-values less than 0.05.

### Co-expression network construction and identification of significant modules

To explore the relevant modules and key genes that contribute to the LS phenotype, WGCNA analysis was conducted on the GSE13355 and GSE14905, separately. Using the “WGCNA” package, a weighted co-expression network was established, and only genes with a standard deviation greater than 0.5 across all samples were included in the analysis. To eliminate outliers in the data, the *goodSampleGenes* function was employed (Supplementary Figures S1A, D). According to the scale-free topology criterion, softPowers value ranging from 1 to 20 were screened out utilizing the *pickSoftThreshold* function, with softPowers of GSE13355 and GSE14905 both found to be 7 (Supplementary Figures S1B, C, E, F). These softPowers values were selected to construct an adjacency matrix, and the most proper β value was chosen to convert the correlation matrix into an adjacency matrix, which was then transformed into a topological matrix. Then, genes were clustered based on TOM using hierarchical clustering method, with the minimum module size set at 80. Similar modules were then merged, and pearson correlation analysis was used to assess the correlation of the merged modules with LS, US, and NS. Finally, those genes closely associated with the LS phenotype was selected for subsequent analyses based on geneModuleMembership >0.8 & geneTraitSignificance>0.5 criteria.

## Identification of key lysosomal genes associated with LS

The key lysosomal genes were identified through intersection of four gene sets as follows:

1) the common DEGs between GSE13355 and GSE14905.
2) the LS phenotype genes of GSE13355 obtained by WGCNA.
3) the LS phenotype genes of GSE14905 obtained by WGCNA.
4) lysosomal genes obtained by GO database.

### Gene set enrichment analysis analysis

GSEA (Subramanian et al., 2005) is a common tool for interpreting high-throughput genomic data by identifying coordinated changes in predefined sets of functionally related genes. “ClusterProfiler” is an R package that provides a user-friendly interface for GSEA analysis. The first step in performing GSEA with “ClusterProfiler” is to annotate the genes of interest. Next, the genes are ranked based on their differential expression, and a pre-defined gene set is tested for enrichment using the hypergeometric test. Finally, the enriched gene sets are visualized barplot implemented by “ggplot2”.

### Biomarker recognition with machine learning algorithm

To reduce the potential bias in target screening, multiple machine learning algorithms were used to obtained the final hub genes in psoriasis classification. Specifically, we firstly used 12 algorithms with variable screening, with different combinations to filter out collinear genes. These algorithms contain Lasso, SVM, glmBoost, Ridge, elastic network (Enet) with different alpha values, RF, stepglm with different modes, partial least squares regression for Cox (plsRcox), generalized boosted regression modeling (GBM), LDA, XGBoost, and NaiveBayes. Then, we extracted the algorithm with the highest AUC value and obtained the robust genes. Subsequently, these robust genes were further fed into RF and Lasso algorithms screening to obtain the final hub genes. In the model training process, the combat_dataset was used to train the model, and the external single dataset was used for model validation. The detailed parameter settings and implementation of the algorithm are as follows.

A total of 108 algorithm combinations were employed to build prediction models utilizing the leave-one-out cross-validation (LOOCV) framework. The Lasso, Enet, and Ridge algorithms were applied using the “glmnet” (Engebretsen and Bohlin, 2019) package. The regularization parameter (λ) was determined through LOOCV, while the L1-L2 trade-off parameter (α) was varied from 0 to 1 (interval = 0.1). The stepwise GLM model was implemented using the “stats” package, utilizing a stepwise algorithm based on the Akaike information criterion (AIC), with the stepwise search direction set to “both,” “backward,” and “forward,” respectively. For the glmBoost model, implementation was carried out using the “mboost” package. The plsRglm model was employed via the “plsRglm” package, utilizing the *cv.plsRglm* function to determine the number of components required, followed by fitting a logistic model using the *plsRglm* function. The GBM model was implemented through the “gbm” package, where the *gbm* function selected the optimal number of trees based on LOOCV error minimization. The SVM model was executed using the “e1071” package, employing a regression approach to enable probability predictions. Additionally, the LDA, XGBoost, and NaiveBayes algorithms were implemented using the “caret”, “xgboost,” and “e1071” packages, respectively.

### Expression validation of hub genes

To explore the relative expression of hub genes in LS, US, and NS groups, we integrated the expression data from GSE13355 and GSE14905 using the *ComBat* function of the “sva” package to eliminate batch effects. The relative expression levels of three identified hub genes were then visualized using boxplots. Furthermore, external datasets, containing GSE30999, GSE34248, GSE41662, and GSE53552 were applied to verify the relative expression of hub genes between LS and US.

### Establishment and evaluation of the nomogram

Nomograms have emerged as useful tools for predicting the survival or occurrence of disease by considering multiple prognostic factors simultaneously. In this study, we constructed predictive nomograms using the “rms” package, incorporating the characteristics of S100A7, SERPINB13, and PLBD1. Calibration curves were used to compare the expected values with the standard values. To evaluate the predictive performance of the nomogram, we utilized the decision curve analysis (DCA) approach. The receiver operating characteristic (ROC) curves were visualized using the “pROC” package.

### Establishment of lysosomal molecular subtypes

The identification of lysosomal molecular subtypes was performed using the “ConsensusClusterPlus” (Wilkerson and Hayes, 2010) package. To further validate the effectiveness of the clustering results, principal component analysis (PCA) was conducted using the “FactoMineR” package. In addition, these molecular subtypes also were validated on several externally independent validation sets, ensuring the robustness of clustering. Based on the previously reported (Charoentong et al., 2017) 28 immune cell gene sets, we used the ssGSEA algorithm to explore the heterogeneity of the immune microenvironment between lysosomal molecular subtypes.

### Gene set enrichment analysis (GSVA)

Functional enrichment analysis was conducted to investigate the heterogeneity among lysosomal subtypes, utilizing the “GSVA” and “limma” packages. Gene sets were obtained from the Molecular Signatures Database (MSigDB), specifically “c2.cp.kegg.v7.4.symbols” and “h.all.v7.4.symbols.gmt.” Statistical significance was determined by assessing absolute t-values of GSVA scores for hallmark pathways and biological functions, with values greater than 1 considered as indicative of significance.

### Estimation of small-molecule compounds

Connectivity Map (CMap) analysis was used to predict small-molecule compounds targeting lysosomal subtypes 1 and 2, following established procedures. In essence, 1309 drug signatures were retrieved from the CMap database (https://clue.io/), and the expression profiles of the top 150 upregulated and 150 downregulated genes were utilized. Subsequently, CMap scores were computed using the eXtreme Sum (XSum) algorithm, and the top five small-molecule compounds with the lowest CMap scores were highlighted.

### Single-cell RNA-seq data quantification and quality control

The raw scRNAseq data were downloaded from GEO database (GSE150672 (Hughes et al., 2020)). Eight samples (three normal samples and five psoriasis samples) were included in our study. To filter out low-quality cells and doublets (2 cells encapsulated in a single droplet), for each sample, cells were removed that had either fewer than 200 unique molecular identifiers (UMIs), over 4,000 or below 200 expressed genes. To filter out dead or dying cells, cells were further removed that had over 20% UMIs derived from mitochondrial genome. This resulted in a total of 18,332 high-quality single-cell transcriptomes in all samples. The total number of UMIs per cell was calculated for the number of UMI sequences of high-quality single cells and genes in the sample. The median normalization process was used to normalize the number of UMIs in each cell to the median of the total UMI of all cells.

### PCA, t-SNE and UMAP reduction

Variable genes were selected using a threshold for dispersion, with z-scores normalized by expression level. The top 2,000 highly variable genes (HVGs) were projected onto a low-dimensional subspace using PCA analysis. The number of principal components (Npcs) were selected based on inspection of the plot of variance explained (Npcs = 30). Single-cell clustering was visualized using 2D uniform manifold approximation and projection (UMAP) or t-Distributed Stochastic Neighbour Embedding (tSNE) for the top 30 principal components with the largest variance explained. The “Clustree” package was utilized to identify the best resolution (resolution = 0.8) in tSNE dimensionality reduction. Finally, 18,332 single cells, including 4,490 normal tissue-derived cells and 13,842 psoriasis tissue-derived cells, were subjected to further analysis. The *FindAllMarkers* function was used to identify the significant markers of each cell cluster. Cell types were assigned to each cluster of cells using well-known markers.

### Benchmark of batch effect correction

To quantitatively assess the absence of batch effects in the single-cell data utilized in this study, we compared two widely employed batch correction algorithms, Seurat and Harmony. For Seurat, we examined two modes: Canonical Correlation Analysis (CCA) and Reciprocal Principal Component Analysis (RPCA). In the CCA model, we segmented the total single-cell objects by sample, followed by standardization and selection of highly variable genes per sample. Subsequently, we employed Seurat’s functions including *SelectIntegrationFeatures*, *FindIntegrationAnchors*, and *IntegrateData* to select features, identify anchors, and integrate the data. Notably, we set the parameters nfeatures to 2000 and ndim to 30, concluding with dimensionality reduction clustering based on the first 30 dimensions of PCA post-correction. For Seurat’s RPCA mode, the calculation steps mirrored those of CCA, with the sole distinction being the setting of reduction = “rpca” during the execution of *FindIntegrationAnchors*. Regarding Harmony integration, we utilized the *RunHarmony* function implemented within Seurat, with batch information designated as samples. Furthermore, to accurately gauge the effectiveness of batch integration and the preservation of biological variations, we employed the Local Inverse Simpson’s Index (LISI) indicator as a metric, encompassing integration-LISI (iLISI) and cell type-LISI (cLISI).

### Statistical analysis

All statistical analysis were performed utilizing R software 4.2.1. Student’s t-test or Wilcoxon were utilized to investigate the difference between two groups. The parameter of correlation analysis between the variables was set as Pearson or Spearman. All statistical *p*-values were two-sided, and *p* < 0.05 or one asterisk (*) was regarded as statistical significance.

## Results

### Feature and functional alterations related to psoriasis

**FIGURE 2:**
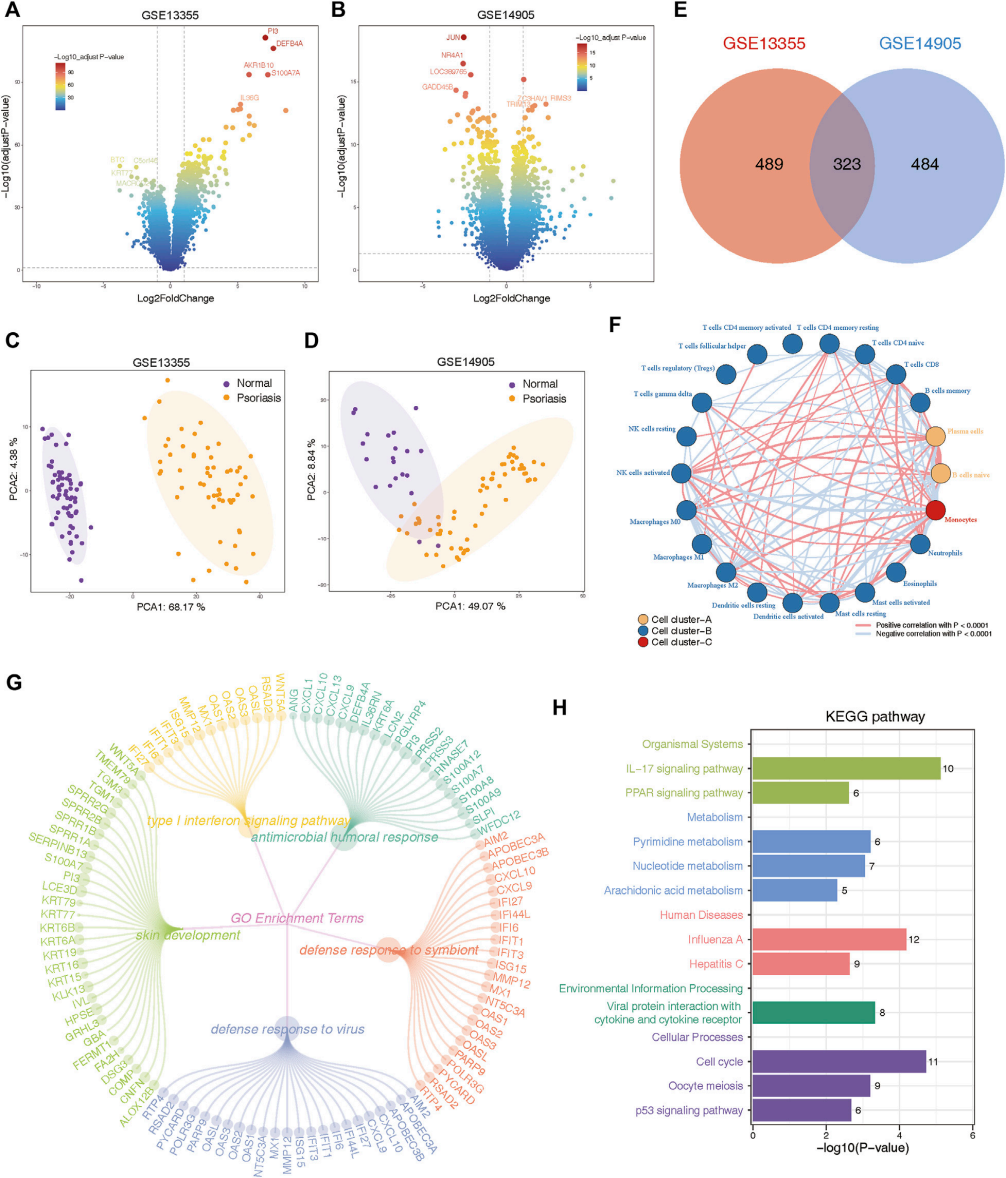
Identification and functional annotation of differentially expressed genes (DEGs) in the expression profiling of GSE13355 and GSE14905 cohorts. **(A)** Volcano plots of GSE13355 depicting the gene expression levels of the DEGs between psoriasis and normal specimens. **(B)** Volcano plots of GSE14905 showing the gene expression levels of the DEGs between psoriasis and normal patients. **(C)** PCA plots showing the gene expression profiling of GSE13355 cohort. **(D)** PCA plots depicting the gene expression profiling of GSE14905 cohort. **(E)** The intersection of DEGs via “limma” package derived from GSE13355 and GSE14905 cohorts, which were shown in the Venn diagram. **(F)** Network plot illustrating interactions among 22 types of immune infiltrating cells. The *p*-values were adjusted using the Benjamini–Hochberg method. Each cell cluster was depicted in a distinct color. Lines connecting the cells represented their interactions, with the thickness indicating the strength of correlation. Positive correlations were denoted by red lines, while negative correlations were represented by blue lines. **(G)** The circle network plot showing the top 5 significantly enriched GO terms, including type Ⅰ interferon signaling pathway, antimicrobial humoral response, skin development and so on. **(H)** Bar plot showing significant pathways calculated by intersected DEGs were involved in organismal systems, metabolism, human disease, environmental information processing, and cellular processes.

In order to avoid artificially introducing correction errors, we conducted differential expression analysis between psoriasis and normal samples on the GSE13355 and GSE14905 cohorts, separately. Consequently, our analysis identified 414 upregulated genes and 393 downregulated genes in GSE14905, and 475 upregulated genes and 337 downregulated genes in GSE13355 datasets (FDR <0.05, abs (log2FC) > 1; Supplementary Table S2). Volcano plots were employed to visualize these DEGs (Figures 2A,B), and PCA analysis also confirmed significant differences between psoriasis and normal samples (Figures 2C,D). Finally, a total of 323 DEGs were commonly identified between GSE13355 and GSE14905 datasets (Figure 2E), and these genes were used to explore whether they were associated with the immune microenvironment. The expression profiles of the aforementioned genes were extracted, and the relative infiltration of immune cells in each sample was inferred using the ssGSEA method. Subsequently, correlations between immune cells were computed, and hierarchical clustering was employed to delineate distinct immune patterns. Our findings reveal the presence of three discernible immune cell patterns (Figure 2F), indicative of pronounced heterogeneity, as evidenced by intricate positive and negative correlations among them. Furthermore, these genes were also subjected to functional enrichment analysis. The GO enrichment analysis revealed that these DEGs were primarily involved in various biological processes, including type Ⅰ interferon signaling pathway, antimicrobial humoral response, skin development, defense response to symbiont and defense response to virus (Figure 2G, Supplementary Table S3). Moreover, KEGG pathways were found to be mainly related to IL-17 signaling pathway, PPAR signaling pathway, metabolism-related pathways and cellular processes (Figure 2H, Supplementary Table S3). These results indicated the presence of metabolic and immune dysregulation in psoriasis.

### Immune microenvironment of psoriasis

In order to gain deep insights into the immune landscape of psoriasis, we employed several computational methods (CIBERSORT and ssGSEA algorithms) to calculate the infiltration scores of immune cells and immune gene sets in different groups. It was found that the infiltration levels of 22 immune cell types and 29 immune gene sets were significantly altered in LS samples compared with NS samples (Supplementary Figures S2A–D). Specifically, we found a higher B cell infiltration compared to NS samples, including that of memory B cell, and plasma cells in patients with LS. Meanwhile, LS patients exhibited higher T cell abundance, including the activated memory CD4^+^ T cell, the T follicular helper cell, the regulatory T cell, and the gamma delta T cell. In addition, natural killer associated cell, macrophage M0, macrophage M1, dendritic cell, mast cell, and neutrophil also had higher infiltration in patients with LS (Supplementary Figure S2B). In order to further validate the results of immune infiltration, another common immune gene set was evaluated between LS and NS samples, which consistent with previous results (Supplementary Figure S2D). It is noteworthy that our analysis revealed a smaller disparity in immune cell abundance between the US group and the NS group compared to the difference observed between the LS group and the NS group (Supplementary Figures S2A, C). This observation suggests that the extent of immune infiltration may influence the progression of the disease. Moreover, we investigated the relative score variances of the hallmark gene set across different groups and observed that the majority of functional terms exhibited significant upregulation in the LS group. Similarly, the disparities observed within the LS group were more pronounced compared to those within the US group (Supplementary Figures S2E, F). Here, we have depicted the heterogeneity of the immune microenvironment in psoriasis, and we planned to search for potential biomarkers for the treatment of psoriasis.

### Identification of LS-associated modules

**FIGURE 3:**
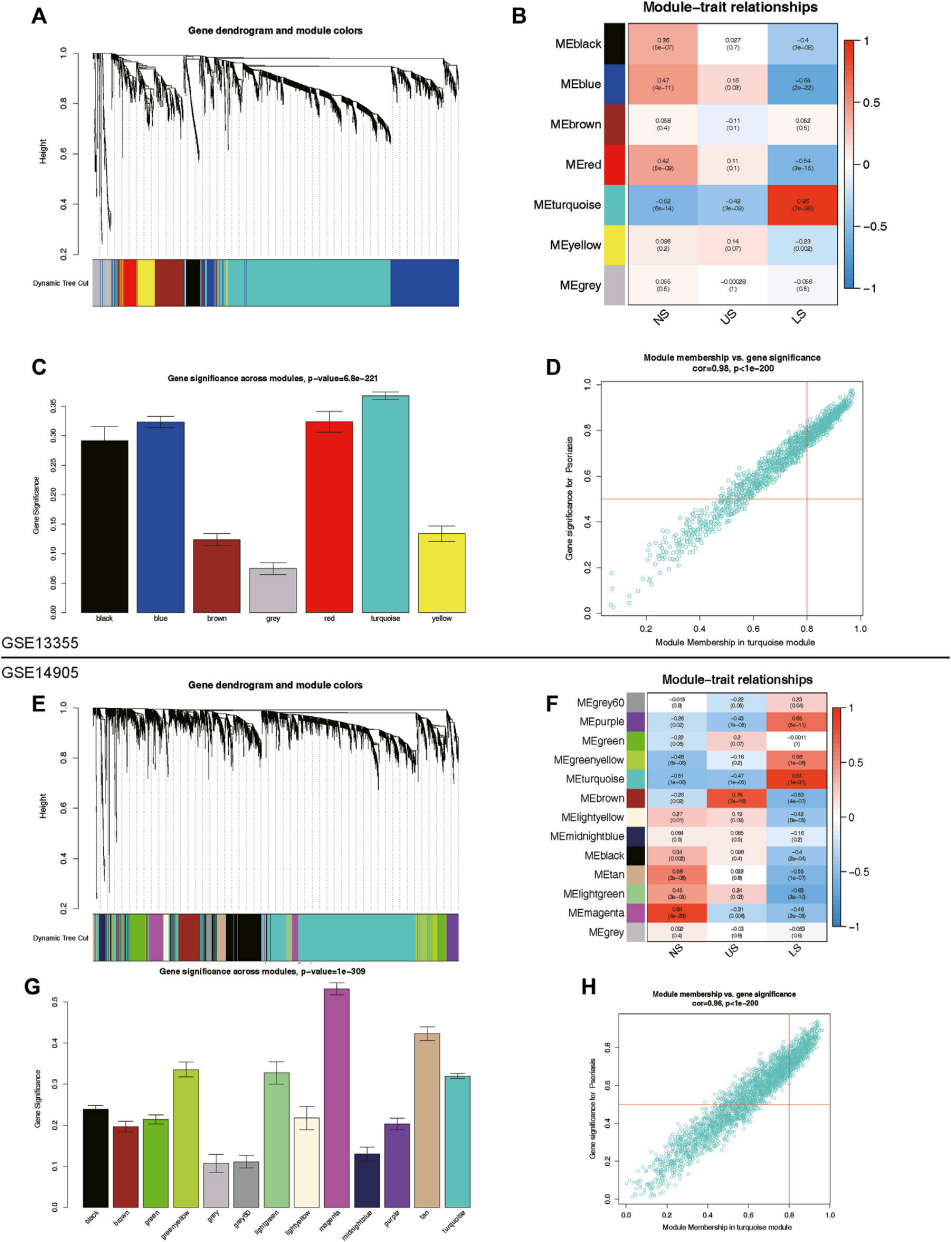
Identification of phenotype co-expression genes. **(A)** Cluster dendrogram of the co-expression network after merging modules in GSE13355 cohort. **(B)** Heatmap of correlation between modules and phenotypes showing that the turquoise module has the highest association with LS. **(C)** Gene significance across modules in GSE13355 cohort. **(D)** Scatter plot of module membership (MM) *versus* gene significance (GS) in turquoise module. Those genes with MM > 0.8 & GS > 0.5 were regarded as hub phenotype Genes. **(E)** Cluster dendrogram of the co-expression network after merging modules in GSE14905 cohort. **(F)** Heatmap of correlation between modules and phenotypes in GSE14905 cohort. **(G)** Gene significance across modules in GSE14905 cohort. **(H)** Scatter plot of MM *versus* GS in turquoise module. LS: Lesion Skin, US: Uninvolved Skin, and NS: Normal Skin samples.

To identify key genes and gene modules related to LS phenotypes, we conducted WGCNA analysis on the basis of the expression profile of GSE13355 and GSE14905. This study focused on genes with a standard deviation greater than 0.5 in all samples, which were consistently clustered to 7 modules in the GSE13355 dataset and 13 modules in the GSE14905 dataset (Figures 3A–C, E–G). After analyzing the person correlation between modules and phenotypes, we found that the turquoise module was highly linked to the LS phenotype, with correlation coefficients of 0.96 and 0.91 in the GSE13355 and GSE14905 datasets, respectively (Figures 3B, C, F, G, *p*-value < 0.001). The module membership (MM) and gene significance (GS) scores of the turquoise module were further used for phenotype gene screening. Those genes with MM > 0.8 and GS > 0.5 were considered statistically significant (Figures 3D,H). As a results, we identified 527 and 602 co-expressed genes linked to the LS phenotype in the GSE13355 and GSE14905 datasets, respectively. These genes were served as phenotypic genes (pheGenes), which provide potential therapeutic targets for psoriasis.

### Identification of lysosomal genes and functional enrichment

**FIGURE 4:**
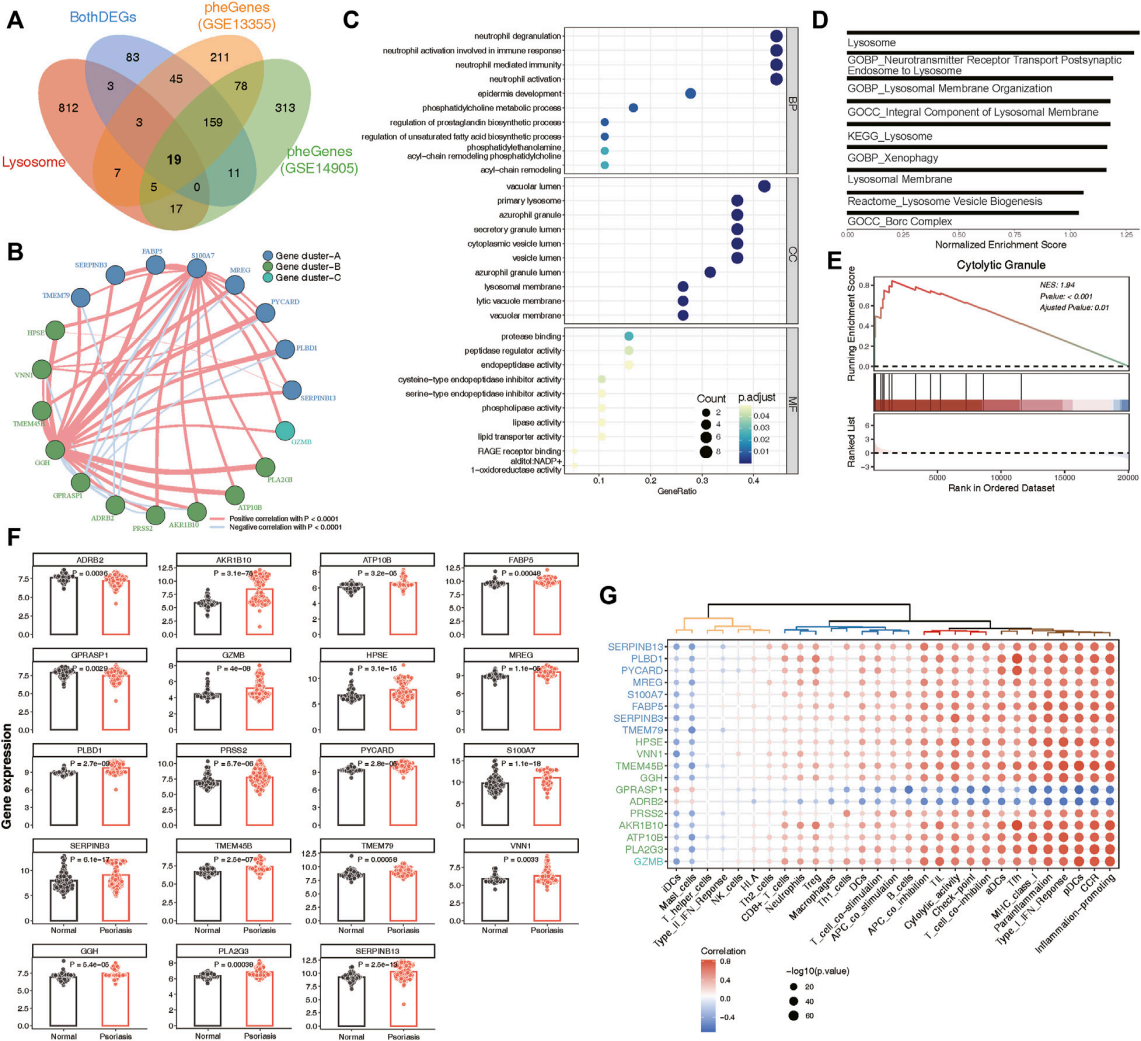
Identification of 19 key lysosomal genes. **(A)** Venn diagram showing 19 common lysosomal genes between BothDEGs, Lysosome, pheGenes_GSE13355 and pheGenes_GSE14905. **(B)** Network plot exhibiting interactions within 19 key lysosomal genes. Each gene cluster was depicted in a distinct color. Lines connecting the genes represented their interactions, with the thickness indicating the strength of correlation. Positive correlations were denoted by red lines, while negative correlations were represented by blue lines. **(C)** The GO enrichment analysis of key lysosomal genes, covering biological progress (BP), cellular components (CC), molecular functions (MF) categories. **(D)** Bar plot showing the significant lysosomal terms. The *x*-axis represented normalized enrichment score of enrichment terms. **(E)** GSEA analysis showing Cytolytic Granule significantly involved in psoriasis samples compared to normal samples. **(F)** Bar chart revealing that the expression of 19 lysosomal genes between psoriasis and normal samples. The statistical comparison between two groups was conducted using the “emmeans” method, allowing for pairwise comparisons of group means. **(G)** Dot plot depicting the correlation between 19 lysosomal genes and 29 immune gene sets, with the size of each circle reflecting the log-standardized *p*-value. Positive correlations were indicated by red color, while negative correlations were represented by blue color. Additionally, the shade of color indicated the correlation coefficient value.

A total of 19 phenotype genes were identified by intersecting the DEG and pheGenes of the GSE13355 and GSE14905 datasets with well-known lysosomal genes (Figure 4A). To assess the correlation among lysosomal genes, we initially illustrated the comprehensive landscape of interactions among 19 lysosomal genes, leading to the identification of three distinct patterns. Within these lysosomal genes, the majority displayed robust synergistic effects (Figure 4B), and functional enrichment analysis revealed that they were primarily involved in several immune reactions, such as neutrophil degranulation and neutrophil-mediated immunity, as well as cell compositions associated with lysosomes, including primary lysosomes and azurophil granules (Figure 4C). Further, GSEA indicated that some lysosome-related pathways might play significant roles in the occurrence and development of psoriasis (Figures 4D,E). One plausible explanation could be the significantly overexpression of the majority of lysosomal genes in the disease group, while only ADRB2 and GPRASP1 exhibited lower expression levels in the disease group compared to the normal group (Figure 4F). Furthermore, we delineated the correlation patterns between the 19 lysosomal genes and 29 immune gene sets. Consistently, the findings underscored significant correlations each other (Figure 4G). This evidence supports the notion that lysosomal functions play vital roles in the pathogenesis of psoriasis, highlighting the potential for these 19 lysosomal genes to serve as therapeutic targets for the disease.

### Identification of candidate hub lysosomal genes via machine learning

**FIGURE 5:**
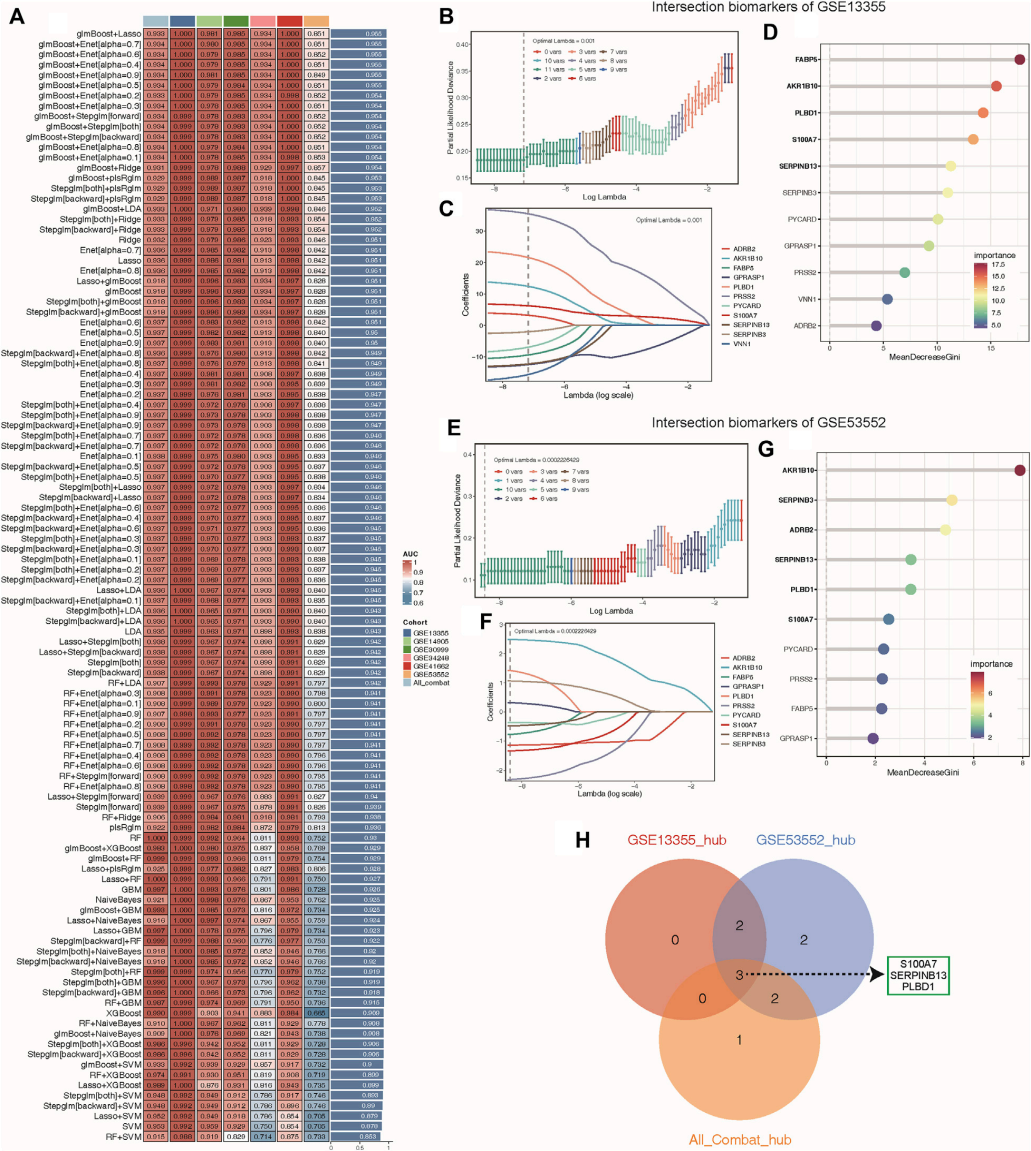
Machine learning identified key diagnosis genes for psoriasis samples. **(A)** Through the LOOCV framework, a total of 108 prediction models were developed and their respective C-index values were computed across all validation datasets. **(B–C, E–F)** A total of 11, 10 key genes were identified with optimal lambda values in the GSE13355 **(B–C)** and GSE53552 **(E–F)** cohorts using Lasso model. **(D, G)** The random forest algorithm ranks the important genes based on MeanDecreaseGini (MDG) scores in the GSE13355 **(D)** and GSE53552 **(G)** cohorts and those genes with important score greater than average were considered as key diagnosis genes (indicated by black bold). **(H)** The overlap of key diagnosis genes calculated by three different datasets is shown in the Venn diagram.

To determine the optimal machine learning model for predicting LS, the combat_datasets was served as the training dataset and each individual dataset as the testing dataset. The expression profiles of 19 lysosomal genes were selected as input variables, and 12 machine learning models, including Lasso, SVM, glmBoost, Ridge, Enet with different alpha values, RF, stepglm with different modes, plsRglm, GBM, LDA, XGBoost, and NaiveBayes, were established to predict outcomes. We fitted 108 kinds of prediction models via the LOOCV framework and further calculated the C-index of each model across all validation datasets (Figure 5A). Consequently, the optimal model was a combination of glmBoost and Lasso with the highest average C-index (0.955), and this model had a dominant C-index in each validation dataset (Figure 5A). Then, we extracted 11 lysosomal genes from this optimal model, which have undergone collinearity filtering. To further identify potential candidate hub genes associated with psoriasis diagnosis, we employed two common and effective machine learning algorithms, containing Lasso regression and RF. The Lasso regression analysis successfully identified 11, 10 and 11 genes that were closely associated with psoriasis in GSE13355, GSE53552 and All_combat datasets, respectively, as evidenced by their nonzero regression coefficients (Figures 5B, C, E, F and Supplementary Figure S3A). Furthermore, the RF algorithm allowed us to evaluate the importance of each gene based on MeanDecreaseGini (MDG) scores, revealing 5, 6 and 6 candidate genes with higher than mean MDG scores in GSE13355, GSE53552 and All_combat datasets, respectively (Figures 5D, G and Supplementary Figure S3A). Finally, the overlapping analyses yielded a total of three lysosomal genes (S100A7, SERPINB13, and PLBD1) as hub candidates for further study (Figure 5H).

### Validation of hub genes at the single-cell level

**FIGURE 6:**
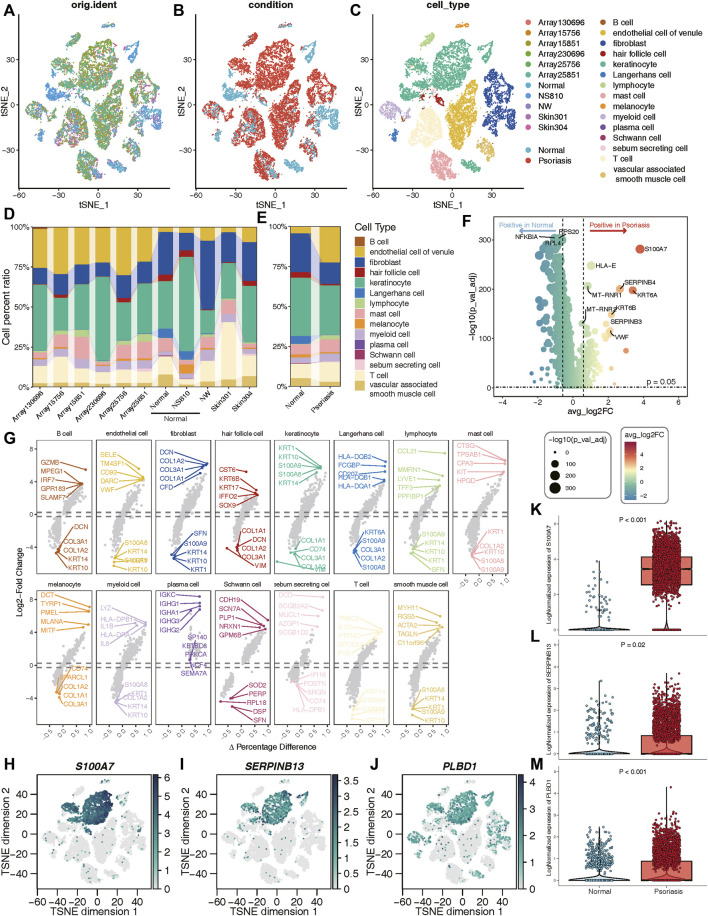
Validation of three hub genes at the single-cell level. **(A–C)** t-SNE reduction of the cells, from left to right: colored by samples; conditions; cell types. **(D, E)** The proportion of different cell types in different samples or conditions. **(F)** Volcano plot showing the DEGs between normal and psoriasis sample. **(G)** The faceted volcanic plot displaying the DEGs in each cell type. **(H–J)** The feature plot showing cell type-specific expression levels of hub lysosomal genes. **(K–M)** The boxplot plot revealing that three hub lysosomal genes were significantly highly expressed in the psoriasis group (*p* < 0.05).

A total of 8 samples (three normal and five psoriasis samples) were involved in this study, which came from GSE150672 cohort. The quality control (QC) criteria are described in Methods (Supplementary Figures S4A–C). Of these, 4,490 single cells originated from normal tissues, while the remaining 13,842 were psoriasis-derived cells (Figure 6B). Each major cell type can be well mixed together according to sample classification (Figure 6A; Supplementary Figure S4D), it suggested that there was no batch effect in this scRNA-seq data. Moreover, we quantitatively demonstrated the absence of batch effects within this dataset (Supplementary Figure S5). We assessed the effectiveness of two commonly used integration methods, Seurat (CCA and RPCA modes) and Harmony, and evaluated the integration efficacy and preservation of biological variation using iLISI and cLISI indicators, respectively (Supplementary Figures S5E, F). Interestingly, Seurat did not exhibit over-correction compared to the original uncorrected data, whereas Harmony demonstrated slight overcorrection (Supplementary Figure S5). Given the importance of avoiding the introduction of artificial errors, we decided not to perform batch effect correction in subsequent analyses. Based on well-known markers, these cells were classified into 15 main cell types (Figure 6C). Notably, when comparing normal cells with psoriasis-derived cells, we found that endothelial, lymphocyte, and T cells were increasingly enriched in psoriasis tissues, while fibroblasts, hair follicle, keratinocyte and langerhans cells mainly existed in normal tissues (Figures 6D,E). These results revealed the heterogeneous landscape among normal and psoriasis samples. We further screening for DEGs between the normal and psoriasis tissues, some common genes (S100A7, SERPINB4) were observed especially expressed in psoriasis tissues (Figure 6F). The faceted volcanic plot further displayed the DEGs in each cell type (Figure 6G). The feature plot and boxplot demonstrated that three hub genes (S100A7, SERPINB13, and PLBD1) were mainly expressed in keratinocyte cells of psoriasis condition (Figures 6H–M). Additionally, we integrated this scRNAseq data and bulk RNAseq data from GSE13355 using the scissor method and found that keratinocytes were most closely related to disease occurrence (Supplementary Figure S6A). These evidences indicate that these three hub genes may play roles in promoting the occurrence and development of psoriasis by regulating the function of keratinocyte cells.

### Potential mechanisms of psoriasis occurrence with Cellchat

**FIGURE 7:**
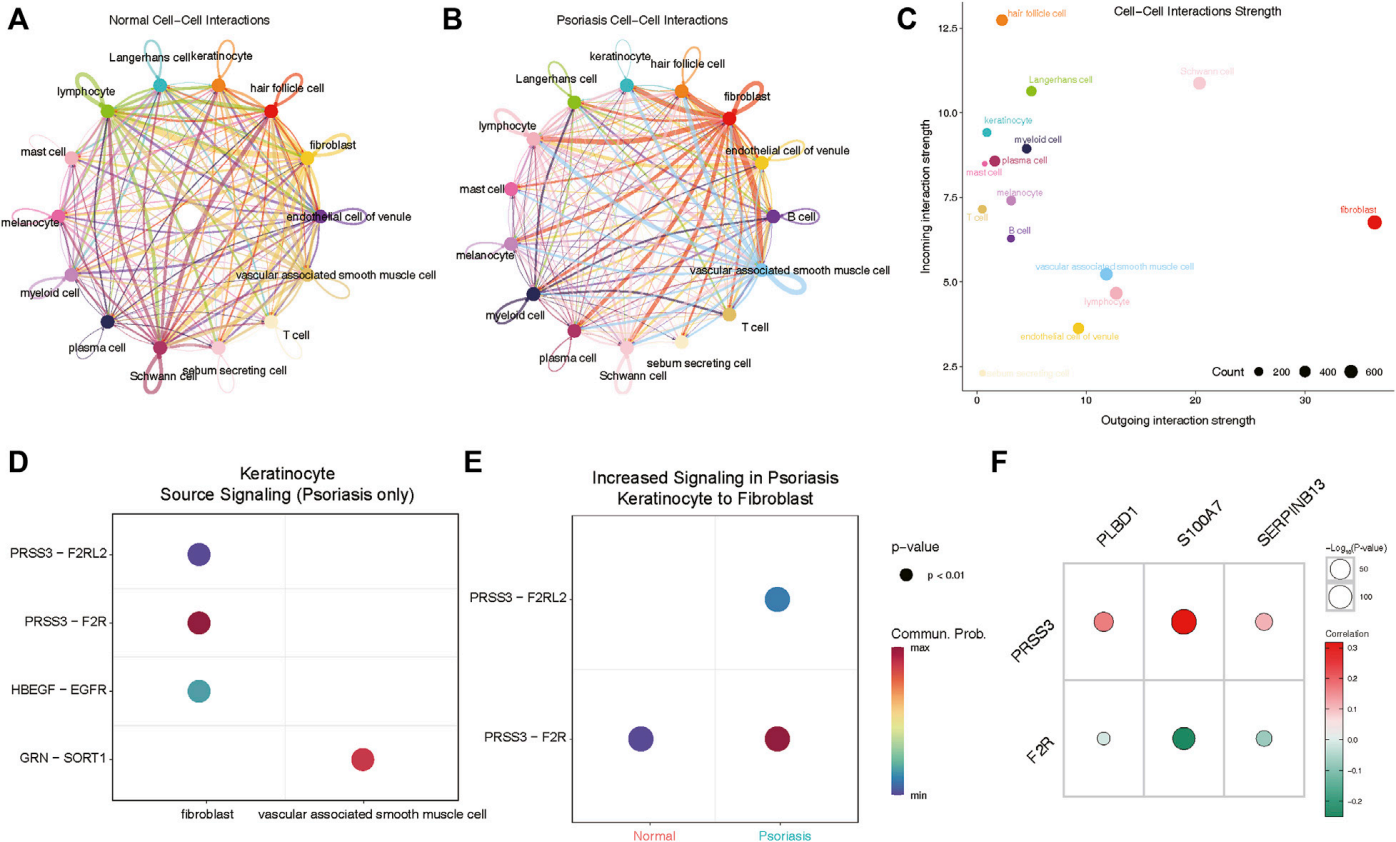
Cellchat analysis indicates keratinocyte communicate with fibroblasts via PRSS3-F2R signaling. **(A, B)** Circle plots of signaling networks of normal **(A)** and psoriasis **(B)**. **(C)** Dot plot showing cell-cell interaction strengths between incoming and outgoing interactions of all cell types. **(D)** Dot plot revealing signaling molecules between keratinocyte and fibroblasts or vascular associated smooth muscle cell. **(E)** PRSS3-F2R signaling between keratinocyte and fibroblasts is highly expressed in psoriasis samples. **(F)** Correlation analysis reveals that three hub lysosomal genes are positively correlated with PRSS3 and negatively correlated with F2R.

We next aimed to investigate whether heightened expression of hub lysosomal genes in keratinocytes coincided with altered intercellular communication in LS. For this purpose, we employed CellChat (Jin et al., 2021), a tool utilizing a database of ligand-receptor interactions to analyze cell-cell communication from scRNA-seq data. Notably, within our scRNA-seq data, intercellular interactions in normal and LS tissues exhibited marked differences (Figures 7A,B). Specifically, robust interactions between lymphocytes, Schwann cells and other cell types were prominent in normal tissues, whereas in LS, fibroblasts and vascular-associated smooth muscle cells displayed strong interactions with other cell types. Additionally, we observed the strongest incoming interactions involving keratinocytes and the strongest outgoing interactions originating from fibroblasts (Figure 7C). Subsequently, we delved into cell-cell interactions in LS, revealing notable communication probabilities between fibroblasts and keratinocytes via PRSS3 binding to F2R and F2RL2 receptors (Figure 7D). Moreover, we observed an overall increase in signaling pairs between fibroblasts and keratinocytes in LS compared to normal tissue, with the PRSS3-F2R signaling axis emerging as the most significant (Figure 7E). Consequently, these genes were subjected to correlation analysis with hub lysosomal genes, revealing strong positive correlations with PRSS3 and negative correlations with F2R (Figure 7F). These evidences suggest a pivotal role for hub lysosomal genes in regulating cell communication between keratinocytes and fibroblasts through the PRSS3-F2R receptor axis.

### Construction of characteristic nomogram for predicting US and LS progression

**FIGURE 8:**
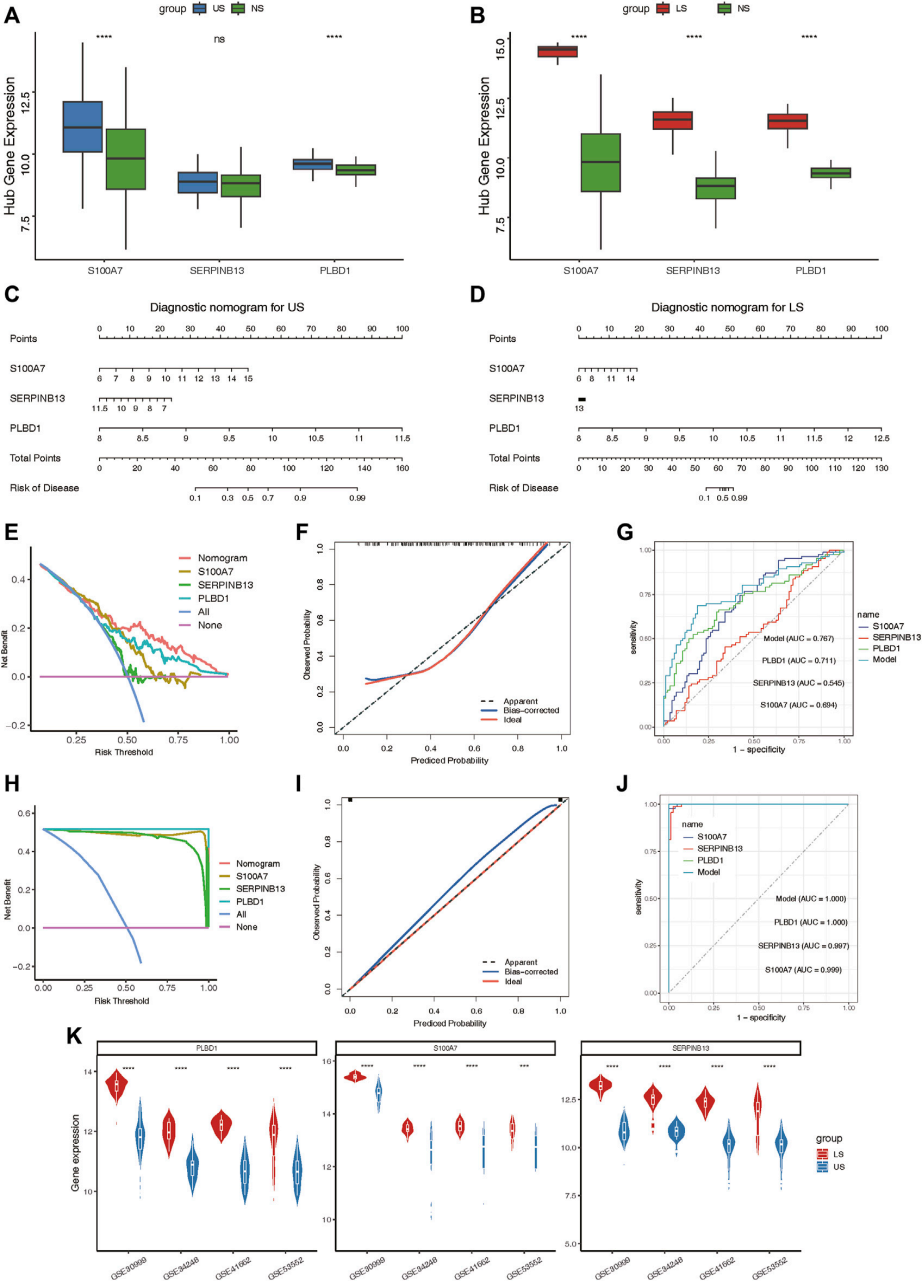
Construction of the nomograms and the diagnosis value assessment. **(A, B)** Boxplot showing the relative expression of three hub lysosomal genes between US and NS, LS and NS. **(C, D)** Nomogram showing three hub lysosomal genes utilized in the diagnosis of patients with US **(C)** and LS **(D)**. **(E)** Calibration curve showing predicted performance of the US model. **(F)** The clinical benefits of the US model evaluated using DCA curves. **(G)** ROC curves were used to evaluate the accuracy of the US model. **(H)** Calibration curve showing predicted performance of the LS model. **(I)** The clinical benefits of the LS model evaluated using DCA curves. **(J)** ROC curves were used to evaluate the accuracy of the LS model. **(K)** Violin plot showing the relative expression of three hub lysosomal genes between US and LS in four independently external RNAseq datasets.

After validating the relative expression of hub lysosomal genes at the single-cell level, we sought to investigate their diagnostic utility through analysis of bulk RNAseq data. Notably, S100A7 and PLBD1 displayed marked upregulation in US samples, while all three hub genes exhibited significant upregulation in LS samples compared to NS samples (Figures 8A,B). In addition, we also found that immune infiltrating cells (Supplementary Figures S7A, B), immune-related gene sets (Supplementary Figures S7C, D), and hallmark gene set (Supplementary Figures S7E, F) was significantly associated with three hub genes, especially in LS samples. Subsequently, we constructed two nomograms for US and LS phenotypes by integrating these characteristic genes (Figures 8C,D). Each gene in the nomogram was assigned a score, enabling the computation of a final score reflecting varying disease risks. Calibration curves for the nomograms accurately estimated the progression of US and LS (Figures 8F,I). Decision curve analysis (DCA) demonstrated that the nomogram provided superior clinical utility in predicting psoriasis occurrence probability at a high-risk threshold of 0–1, compared to individual curves for S100A7, SERPINB13, and PLBD1 (Figures 8E,H). Furthermore, ROC curve analysis affirmed the model’s precision in predicting psoriasis occurrence probability, outperforming single independent predictive factors (Figures 8G,J). Additionally, the heightened expression of these three hub genes in LS phenotypes relative to US phenotypes was validated across four external RNAseq datasets (Figure 8K). These findings underscore the potential of identified hub lysosomal genes and nomograms as robust diagnostic tools for psoriasis, offering valuable insights for clinical decision-making.

### Construction of two molecular subtypes of psoriasis

**FIGURE 9:**
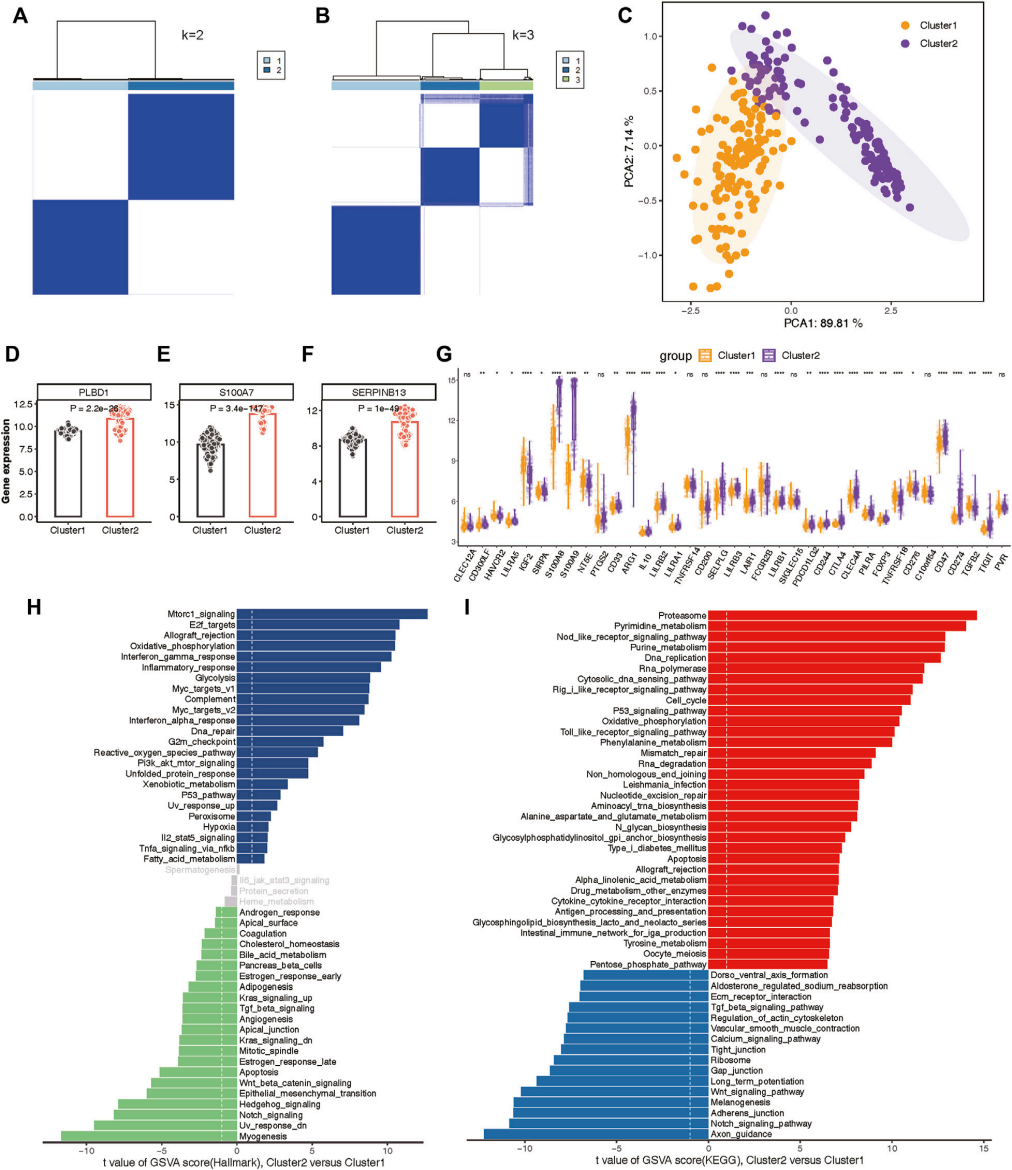
Construction of two molecular subtypes based on three hub lysosomal genes in the integrated datasets. **(A, B)** Consensus matrix heatmap when k = 2 **(A)** and k = 3 **(B)**. **(C)** PCA analysis showing the significant difference between Cluster 1 and Cluster 2. **(D–F)** Bar chart showing the relative expression of three hub lysosomal genes between Cluster 1 and Cluster 2. **(G)** Boxplot showing relative expression of immune check points. **(H, I)** Differential analysis of hallmark terms **(H)** and enriched biological pathways **(I)** was conducted among distinct lysosomal subtypes, ranked by t values of GSVA scores. t-value greater than 1 was considered statistically significant.

To delineate the expression patterns of hub lysosomal genes in LS, we applied the consensus clustering algorithm to group disease samples based on the expression profiles of the three hub genes. The consensus matrix served as a similarity matrix to define final subtypes. Upon analyzing the consensus clustering results and assessing the cluster stability, we determined that k = 2 was the optimal value, resulting in the classification of samples into Cluster 1 and Cluster 2 (Figures 9A,B; Supplementary Figure S8A). PCA analysis further highlighted the pronounced differences between Cluster 1 and Cluster 2 (Figure 9C). As anticipated, substantial heterogeneity was observed in the expression of the three hub lysosomal genes between these subtypes (Figures 9D–F). Additionally, significant change in the expression of immune checkpoint molecules (Figure 9G), immune infiltrating cells (Supplementary Figure S8B), and hallmark gene sets (Supplementary Figure S8C) was evident between these subtypes. The external independent validation dataset confirmed the clustering robustness and repeatability of the two subtypes, whether it is the integrated dataset or the individual datasets (Supplementary Figure S9).

Subsequently, we characterized enriched biological functions and signaling pathways using hallmark and KEGG gene sets from the MSigDB database and estimated the score of each patient using GSVA. In Cluster 2, biological functions related to immune response, such as Interferon gamma response, Inflammatory response, and Complement activation, were notably enriched (Figure 9H). Metabolism-related functions, including Oxidative phosphorylation, Glycolysis, and Xenobiotic metabolism, were also highly enriched in Cluster 2. Conversely, biological functions in Cluster 1 primarily involved Notch signaling, Hedgehog signaling, Wnt/β-catenin signaling, and TGF-β signaling (Figure 9H). Moreover, the pathways associated with lysosomal Cluster 2 enrichment were consistently linked to metabolism and immunity, encompassing metabolism-associated pathways and Antigen processing and presentation. While lysosomal Cluster 1 exhibited activation of pathways such as the Notch signaling pathway and Wnt signaling pathway (Figure 9I).

### Immune and metabolic profiling of lysosomal subtypes in psoriasis

**FIGURE 10:**
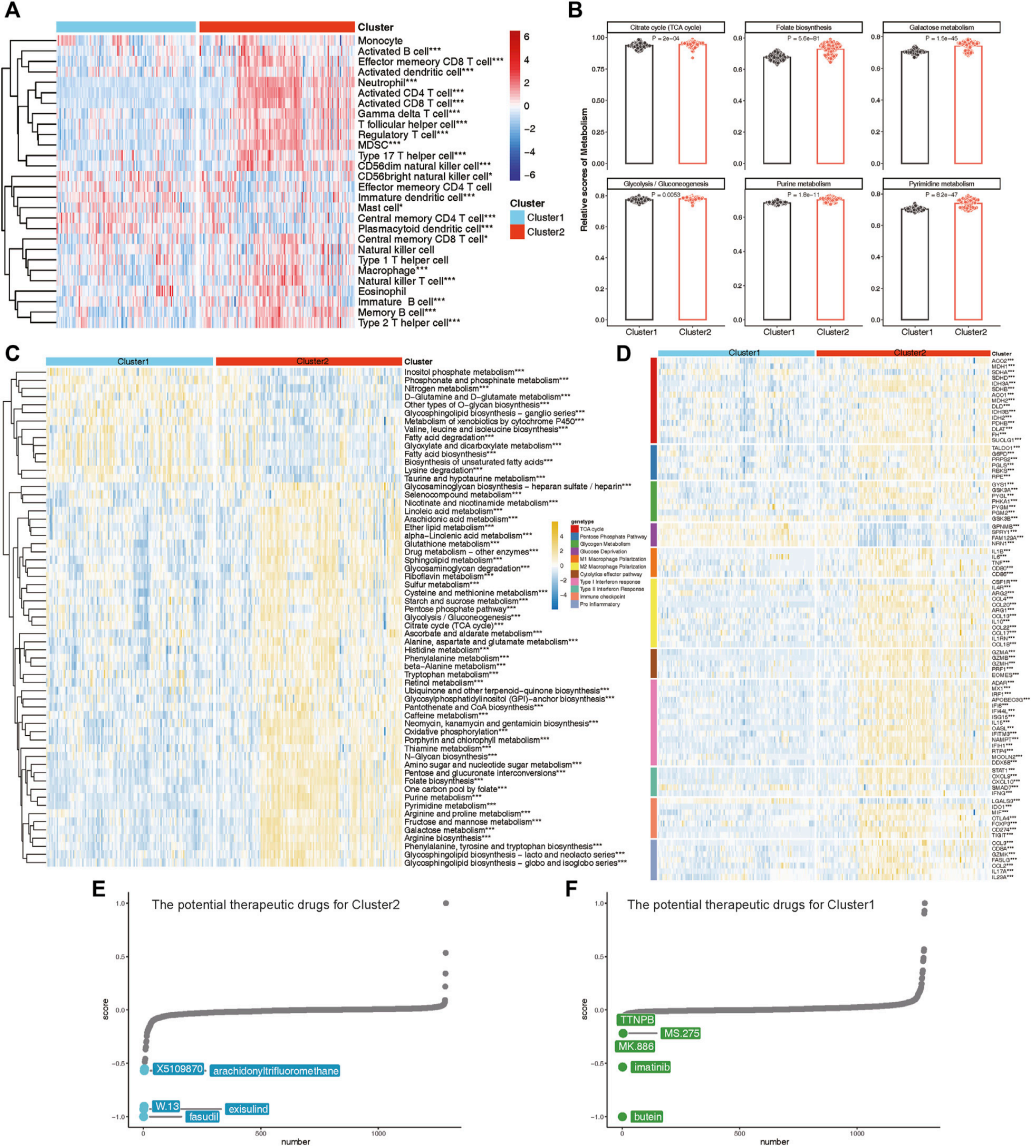
The immune and metabolic characteristics of distinct lysosomal subtypes. **(A)** Heatmap revealing the relative ssGSEA scores of 28 immune cell subsets across different subtypes. **(B)** Bar chart showing relative scores of highlighted metabolic gene sets between subtypes. **(C)** Heatmap reveals the significant metabolic gene sets across different subtypes. **(D)** Heatmap indicating the differences in immune- or metabolism-related genes between Cluster 1 and Cluster 2. **(E, F)** CMap analysis showing the potential therapeutic compounds for Cluster 2 **(E)** and Cluster 1 **(F)**, respectively. *p < 0.05, **p < 0.01, and ***p < 0.001.

To elucidate the immune and metabolic distinctions and their interplay between these lysosomal subtypes, we initially compared differences in 28 immune cell subsets within each subtype. Patients in Cluster 2 exhibited significantly higher infiltration of various T cell subsets, including Natural killer T cells, Effector memory CD8 T cells, Activated CD4 T cells, Activated CD8 T cells, Gamma delta T cells, T follicular helper cells, Regulatory T cells, Type 2 T helper cells, Type 17 T helper cells, and Central memory CD8 T cells, compared to those in Cluster 1 (Figure 10A). Moreover, multiple B cell subsets, including immature B cells, activated B cells, and memory B cells, as well as CD56dim natural killer cells, macrophages, MDSCs, and neutrophils, displayed higher enrichment scores in lysosomal Cluster 2 (Figure 10A).

Furthermore, we explored specific metabolic patterns between subtypes and observed distinct metabolic signatures. Notably, Folate biosynthesis, Galactose metabolism, Purine metabolism, and Pyrimidine metabolism were significantly enriched in Cluster 2, whereas Fatty acid biosynthesis and Lysine degradation were notably enriched in Cluster 1 (Figures 10B,C).

As evidence of verification, we also evaluated the expression of immune regulatory and metabolic genes in each subtype (Figure 10D). In Cluster 2, nearly all immune markers related to M1 Macrophage Polarization, M2 Macrophage Polarization, Cytolytic effector pathways, Type I Interferon response, Type II Interferon Response, Immune checkpoints, and Pro-inflammatory factors were consistently highly expressed. Concurrently, lysosomal Cluster 2 also exhibited enhanced expression of metabolic genes involved in the TCA cycle, Pentose Phosphate Pathway, and Glycogen Metabolism (Figure 10D). Notably, patients in Cluster 1 demonstrated specific upregulation of metabolic genes associated with Glucose Deprivation compared to Cluster 2. Taken together, these findings led to the identification of Cluster 1 as a signaling-activation subtype and Cluster 2 as a mixed subtype characterized by immune and metabolic interplay.

Finally, we investigated potential therapeutic drugs targeting signaling-activation subtype 1 and immune-metabolism subtype 2 using CMap analysis. A lower CMap score for a small molecule compound suggests a higher likelihood of it being effective in treating the disease. In immune-metabolism subtype 2, fasudil, exisulind, W.13, arachidonyltrifluoromethane, and X5109870 emerged as the top five small-molecule compounds with the lowest CMap score (Figure 10E). Conversely, in signaling-activation subtype 1, butein, imatinib, MK.886, MS.275, and TTNPB were identified as relevant small-molecule compounds (Figure 10F). CMap analysis revealed that fasudil and butein were the most promising therapeutic drugs for targeting immune-metabolism subtype 2 and signaling-activation subtype 1, respectively.

## Discussion

Psoriasis is a complex immune-mediated systemic inflammatory disorder (Masson et al., 2020). The pathogenesis of psoriasis involves inflammatory mechanisms controlled by several key genes. Research has found that dyslipidemia and immune disorders might increase the risk of psoriasis (Aksentijevich et al., 2020). Moreover, lysosomal dysfunction has been increasingly associated with both disease and aging (Pu et al., 2016; Platt et al., 2018). Despite researchers linking lysosomes to psoriasis since the 1970s and several studies recently identifying biomarkers for psoriasis, none have yet identified lysosome-related biomarkers. Our preliminary research findings indicate a close association between lysosomal pathways and the onset and pathological progression of psoriasis. Consequently, this study focuses on lysosome-related genes and employs various analytical methods to screen for potential targets capable of predicting disease occurrence and effectively treating psoriasis.

In this study, three hub lysosomal genes (S100A7, SERPINB13, and PLBD1) were determined as potential diagnostic targets of psoriasis in combination with DEGs analysis, WGCNA and multiple machine learning methods (Figure 5). Specifically, our study suggests that S100A7, one of the identified genes, may serve as a promising therapeutic target with few undesired side effects due to its narrow spectrum of biological effects (D'Amico et al., 2016). Various treatments, including calcipotriol, a vitamin D analog, and narrow-band UVB phototherapy, have been found to reduce S100A7 expression and interfere with the inflammatory loop, potentially leading to improved psoriasis outcomes (Hegyi et al., 2012; Batycka-Baran et al., 2015). Furthermore, recent findings suggest that the anthocyanidin delphinidin may also suppress S100A7 expression (Chamcheu et al., 2015), highlighting its potential as a new therapeutic approach. By downregulating S100A7 expression, this may decrease the production of TNF, IL-6, and IL-8 from neutrophils, which are known to play a crucial role in the pathogenesis of psoriasis (D'Amico et al., 2016). As a cross-class specific serine protease inhibitor of Cat L, SERPINB13 was found to be mainly confined to the basal layer in normal skin samples (Bylaite et al., 2006). However, in diseased skin, SERPINB13 was significantly overexpressed and redistributed, indicating its potential involvement in the pathogenesis of psoriasis. Interestingly, our study also revealed that SERPINB13 is a key regulator of psoriasis, as evidenced by its rapid decrease in expression following dithranol treatment in psoriatic patients (Benezeder et al., 2020). Despite the lack of previous research investigating the relationship between PLBD1 and psoriasis, our study has uncovered evidence suggesting that this gene may hold significant promise as a target for the treatment of psoriasis. Our findings add to the growing body of knowledge surrounding the underlying mechanisms of this complex immune-mediated disease and highlight the potential for novel therapeutic approaches. By targeting PLBD1, we may be able to develop more effective and precisely tailored treatments for psoriasis that improve patient outcomes. Further investigation is warranted to fully elucidate the role of PLBD1 in psoriasis and its potential as a therapeutic target. All in all, these findings not only provide insights into the pathogenesis of psoriasis but also pave the way for more targeted and effective treatments for patients with this complex immune-mediated disease.

Aberrant activation of immune cells has been identified as a key factor in psoriasis development. The increased infiltration of dendritic cells, macrophages, neutrophils, and NK cells is a prominent feature of psoriasis (Lowes et al., 2014), with each cell type playing a crucial role in the pathogenesis of disease. Dendritic resting cells contribute to T lymphocyte activation and cytokine/chemokine production (Chiricozzi et al., 2018), while macrophages are critical for lymphocyte activation and proliferation, facilitating the inflammatory process (Lorthois et al., 2017). Neutrophils, a primary origin of pro-inflammatory mediators like IL-17, have also been implicated in psoriasis progression (Chiricozzi et al., 2018). Furthermore, NK cell infiltration and cytokine release are believed to play a role in regulating immune responses in psoriasis (Kucuksezer et al., 2021). In this study, we investigated the relevance between characteristic targets and immune cells in psoriasis. Our findings indicate that S100A7, SERPINB13, and PLBD1 may contribute to the inflammatory process of psoriasis by impacting macrophages infiltration, especially in LS samples (Supplementary Figure S6). Furthermore, these three genes may also link to regulate immune responses by affecting activated NK, T cell and neutrophils infiltration. Our study provides new insights into the complex immunopathogenesis of psoriasis, potentially aiding in the development of targeted therapeutic strategies.

With the development of single-cell technology, we have gained the ability to identify and validate hub genes at the single-cell level, providing insights into their potential mechanisms underlying disease onset and progression. In this study, we observed that three hub lysosomal genes (S100A7, SERPINB13, and PLBD1) exhibit predominant expression in keratinocytes, with their relative expression levels being notably elevated in diseased tissues compared to normal tissues (Figure 6). Additionally, our investigation revealed that keratinocytes primarily interact with fibroblasts, with the PRSS3-F2R ligand-receptor pair serving as a key mediator in this cell-cell interaction (Figures 7A–E). Correlation analysis further demonstrated a robust interaction between PRSS3 and the hub genes (Figure 7F). Consequently, we reasonably speculate that these three hub genes play a pivotal role in regulating cell-cell communication between keratinocytes and fibroblasts through the PRSS3-F2R axis, thus highlighting their potential significance in disease pathogenesis.

This study also offers promising insights into the molecular subtypes of psoriasis, with implications for the personalized treatment of patients. By utilizing a consensus clustering approach, we were able to identify two distinct molecular subtypes, Cluster 1 and Cluster 2, based on the expression profiling of three key lysosomal genes (Figure 9). Notably, our analysis revealed that the Cluster 2 subtype represents a mixed subtype characterized by immune and metabolism, which exhibited immune, metabolic phenotypes and more pronounced immune checkpoint or metabolic expression compared to the Cluster 1 subtype, underscoring the importance of immune and metabolic dysregulation in the pathogenesis of psoriasis (Figures 9, 10). For Cluster 1, it may be a signaling-activation subtype, showing higher enrichment levels of disease signaling pathways, such as: Notch signaling, Wnt/β-catenin signaling, and TGF-β signaling pathways (Figures 9H,I). This classification strategy enabled us to capture the intricate immune landscape of different psoriasis groups, thereby enhancing the accuracy of early diagnosis and intervention of psoriasis treatment.

Nevertheless, it is important to note that this study has certain limitations. For instance, given that this study relied on publicly available datasets, further validation through prospective samples is warranted for experimental confirmation. Additionally, the sample size utilized for identifying molecular subtypes or training machine learning algorithms was relatively small, underscoring the need for a larger LS sample size for robust validation. Finally, the inability to comprehensively evaluate distinct subtypes of LS patients stems from the absence of critical clinical characteristics such as medication responsiveness, smoking and drinking habits, and prior therapies.

## Conclusion

Our study successfully identified and validated three hub lysosomal genes (S100A7, SERPINB13, and PLBD1) at the bulk and single-cell levels, which were served as potential predictors of psoriasis occurrence. Further, these hub genes were likely involved in the cell-cell communication process between keratinocytes and fibroblasts, and were influenced by the PRSS3-F2R. Furthermore, we proposed a new molecular classification that distinguished different subtypes in psoriasis samples. The significance of these findings lies in the potential for developing more targeted immunotherapy for psoriasis patients. Collectively, it offers hope for improved outcomes and quality of life for psoriasis patients.

## Data Availability

The original contributions presented in the study are included in the article/Supplementary Material, further inquiries can be directed to the corresponding author.

## Abbreviations

GEO, Gene Expression Omnibus; DEGs, differentially expressed genes; WGCNA, weighted gene co-expression network analysis; ROC, receiver operating characteristic; ssGSEA, single-sample gene set enrichment analysis; GO, gene ontology; KEGG, Kyoto encyclopedia of genes and genomes; GSEA, gene set enrichment analysis; BP, biological progress; CC, cellular components; MF, molecular functions; MDG, mean decrease gini; LS, Lesion Skin; US, Uninvolved Skin; NS, Normal Skin.
