# Supplementary figures and images for: Single-cell and bulk RNAseq unveils the immune infiltration landscape and targeted therapeutic biomarkers of psoriasis
Source: Front Genet. 2024 Apr 18;15:1365273. doi: 10.3389/fgene.2024.1365273 (PMC11063342; doi:10.3389/fgene.2024.1365273)

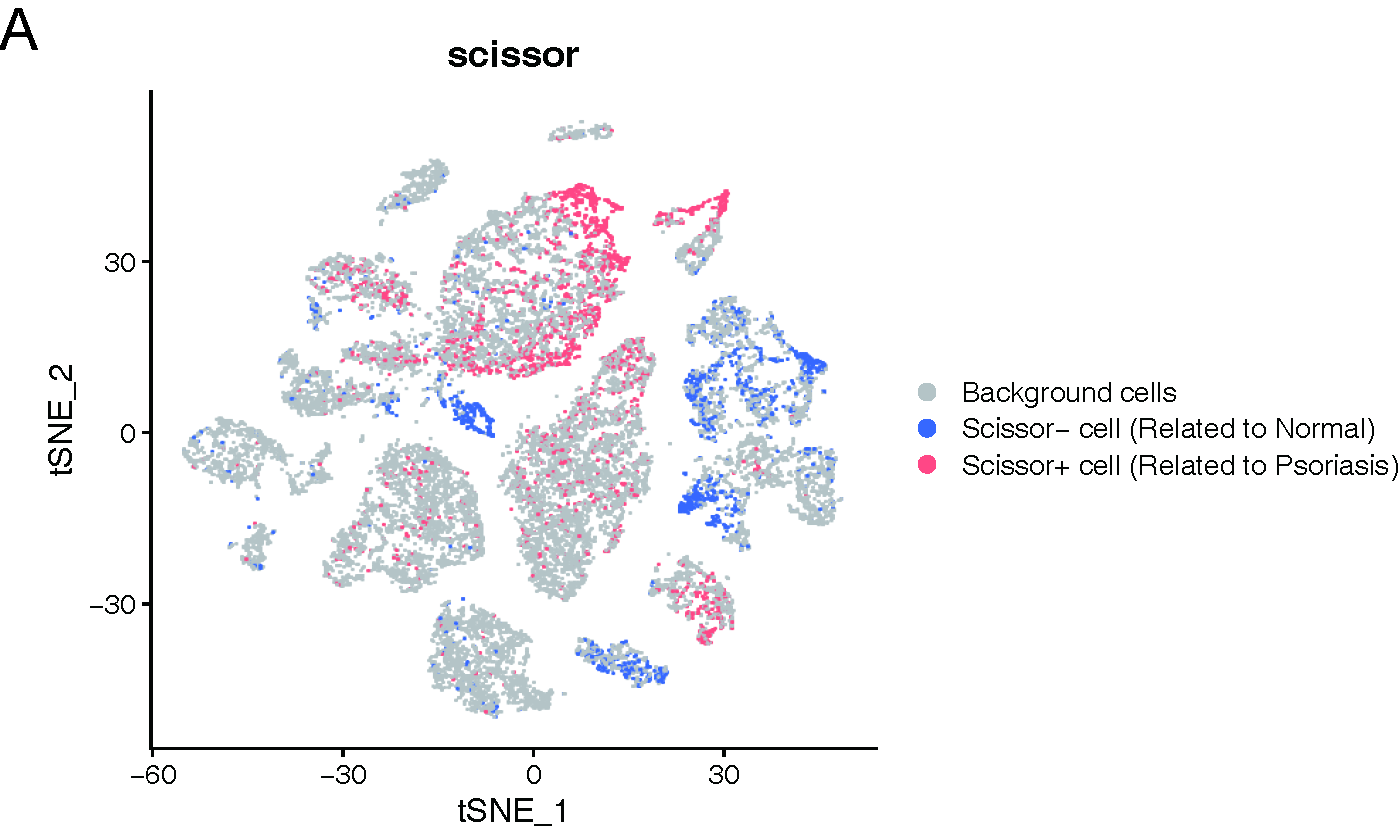

Supplement: Supplementary file 3 [file Image6.TIF]

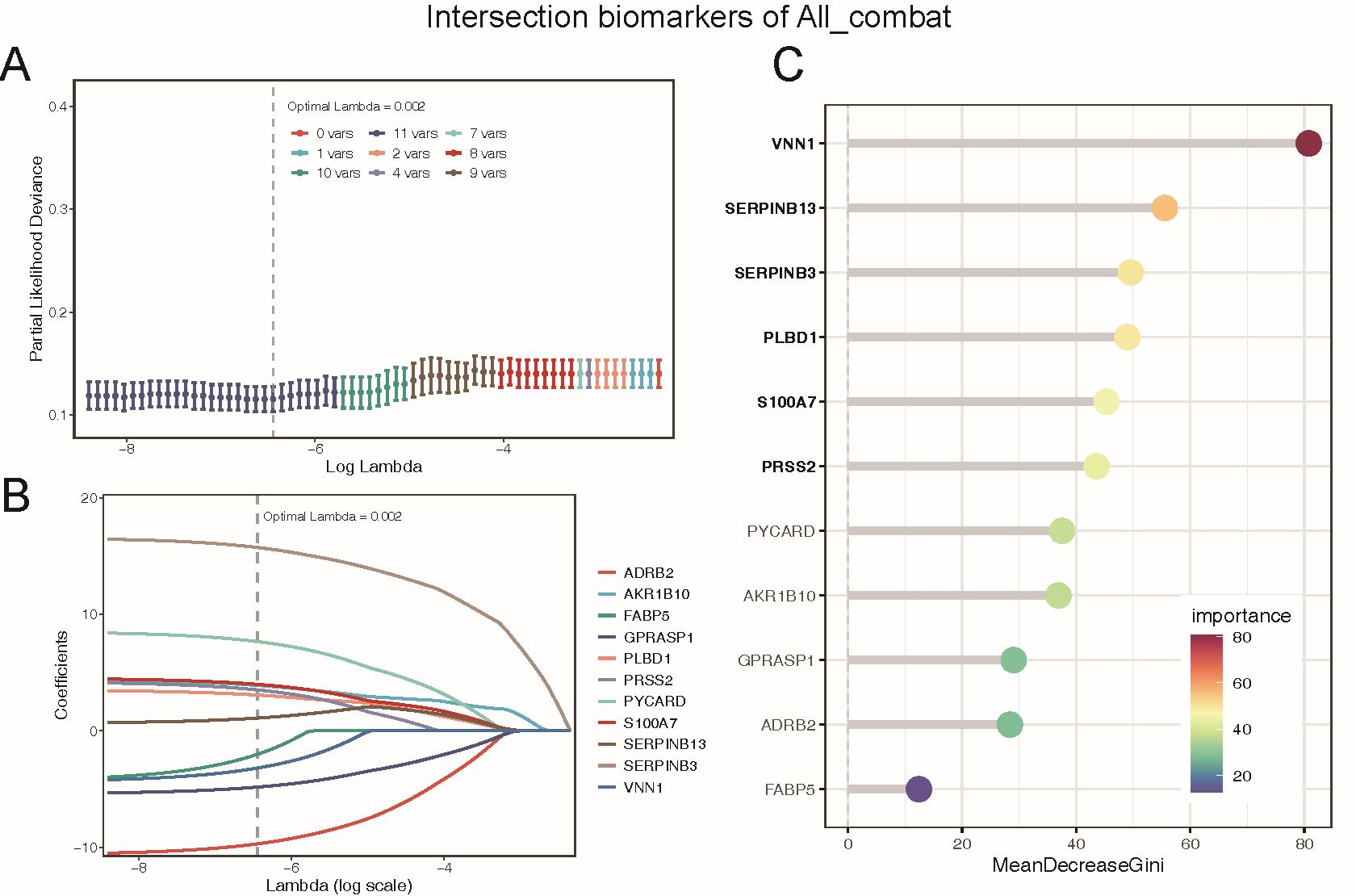

Supplement: Supplementary file 4 [file Image3.TIF]

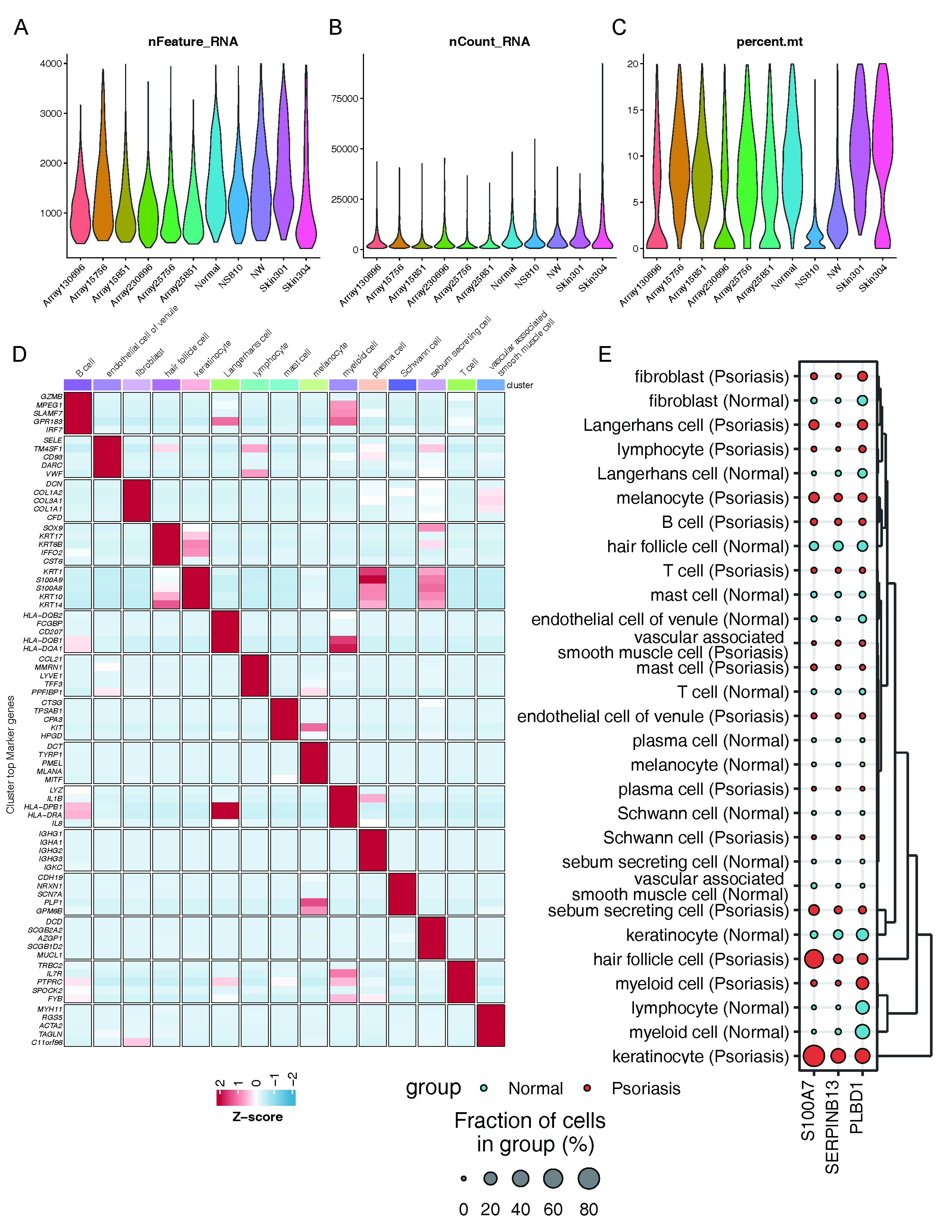

Supplement: Supplementary file 5 [file Image4.TIF]

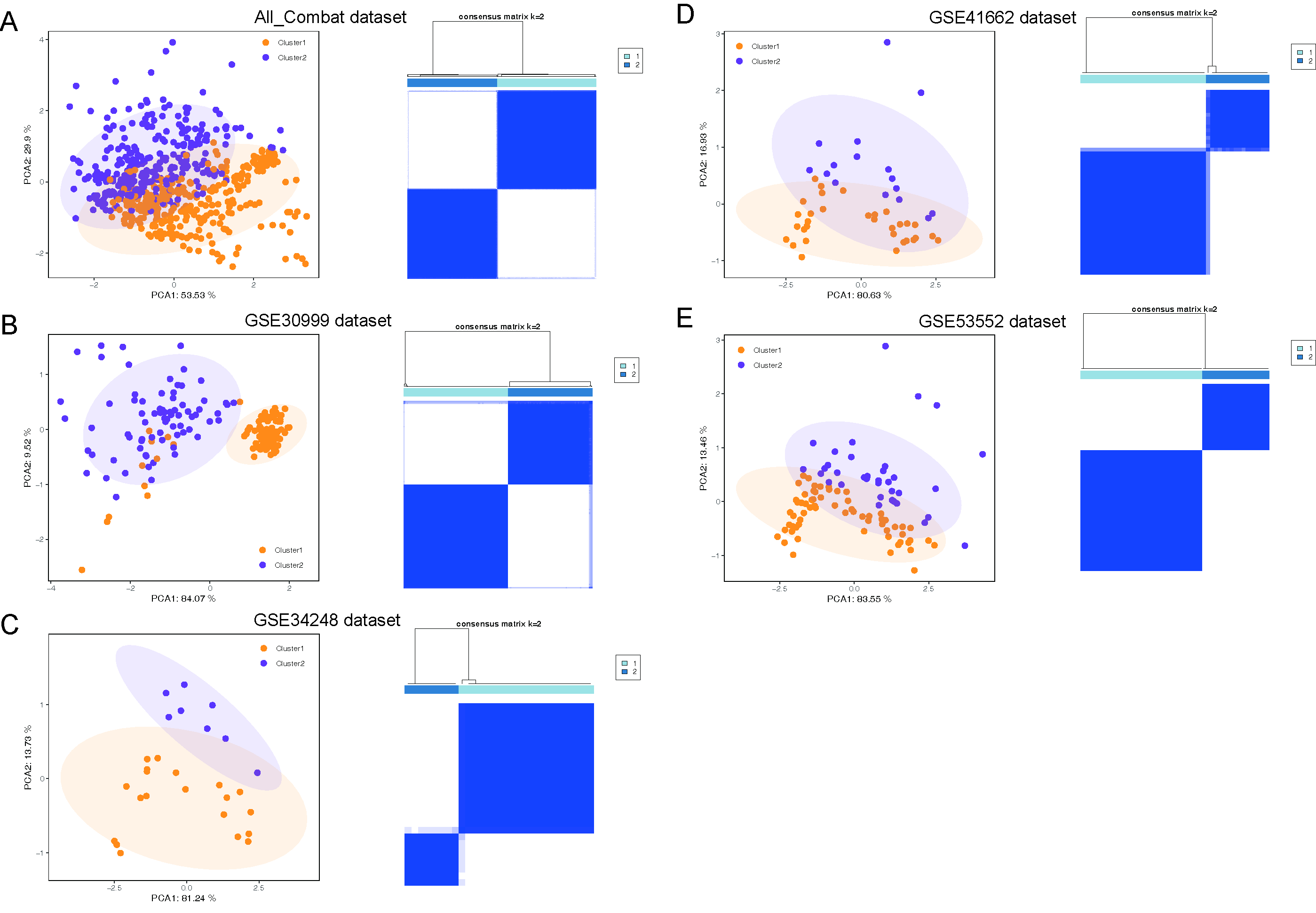

Supplement: Supplementary file 6 [file Image9.TIF]

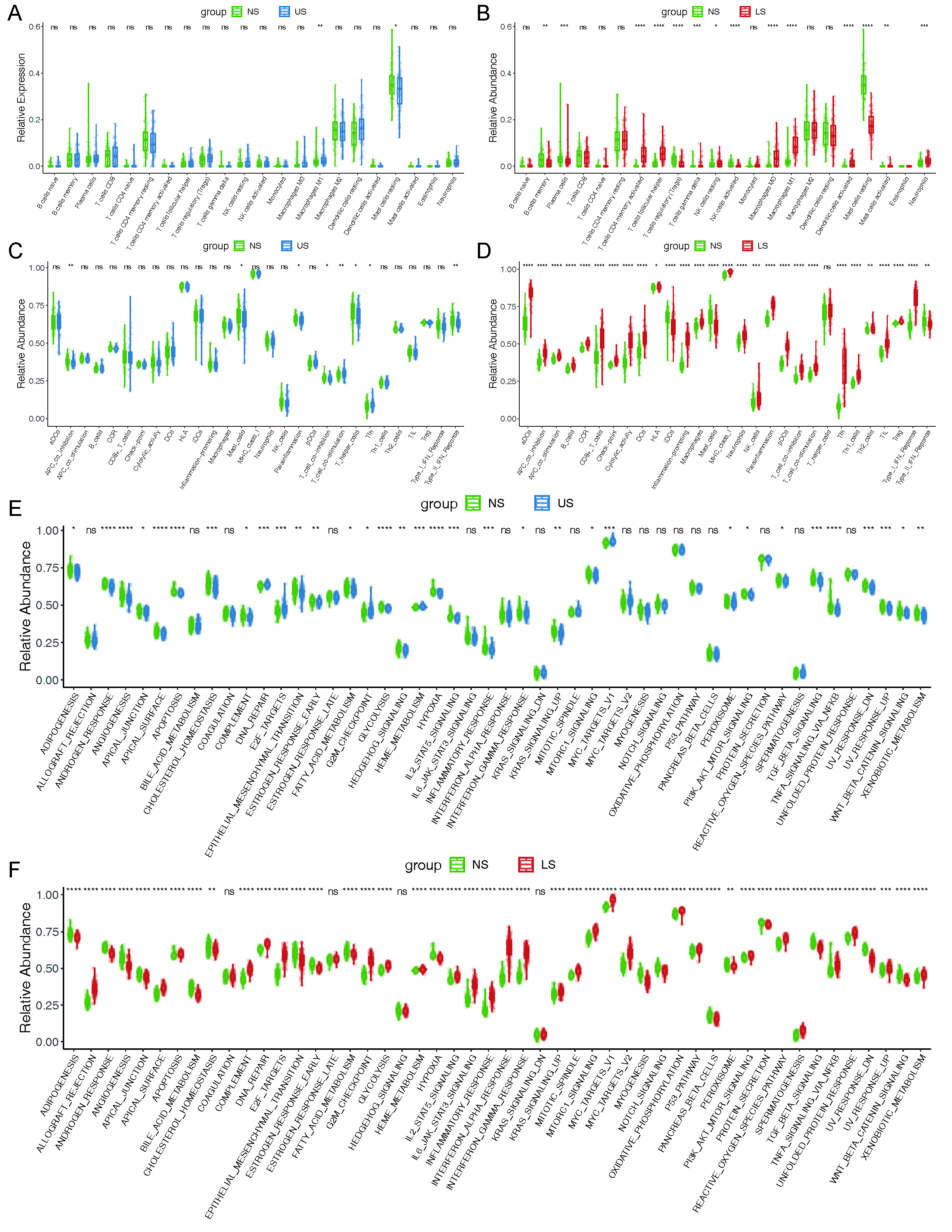

Supplement: Supplementary file 7 [file Image2.TIF]

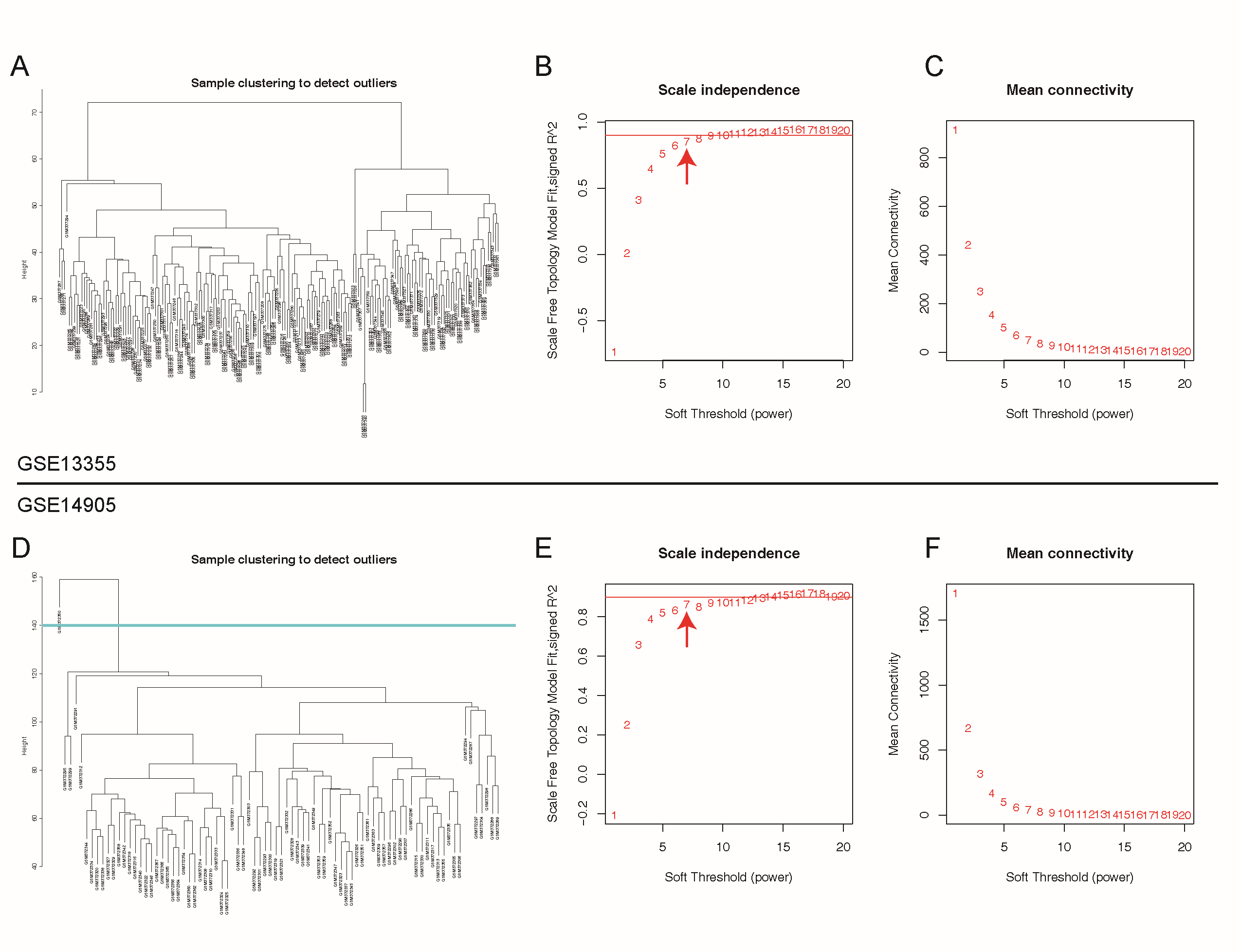

Supplement: Supplementary file 8 [file Image1.TIF]

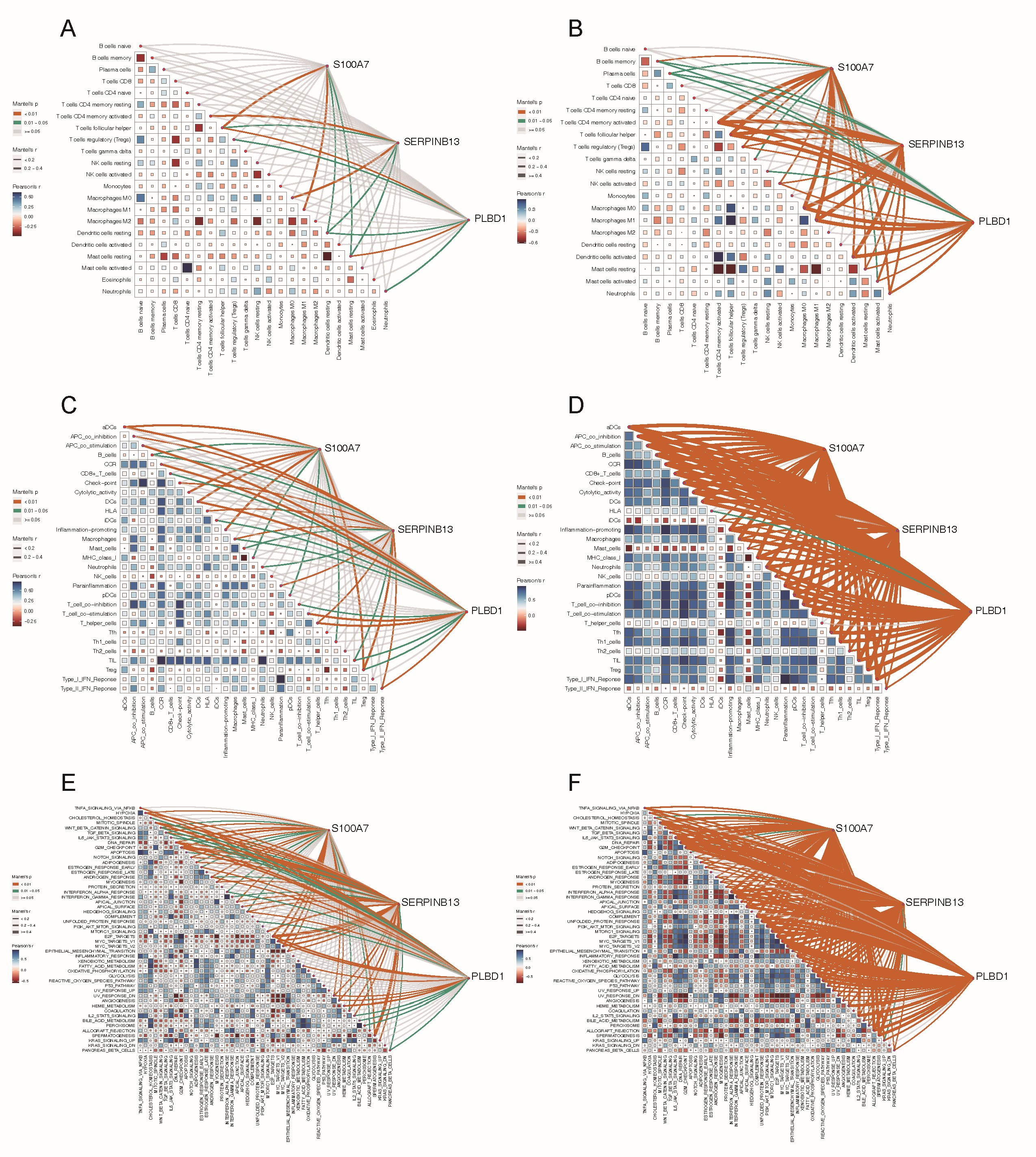

Supplement: Supplementary file 9 [file Image7.TIF]

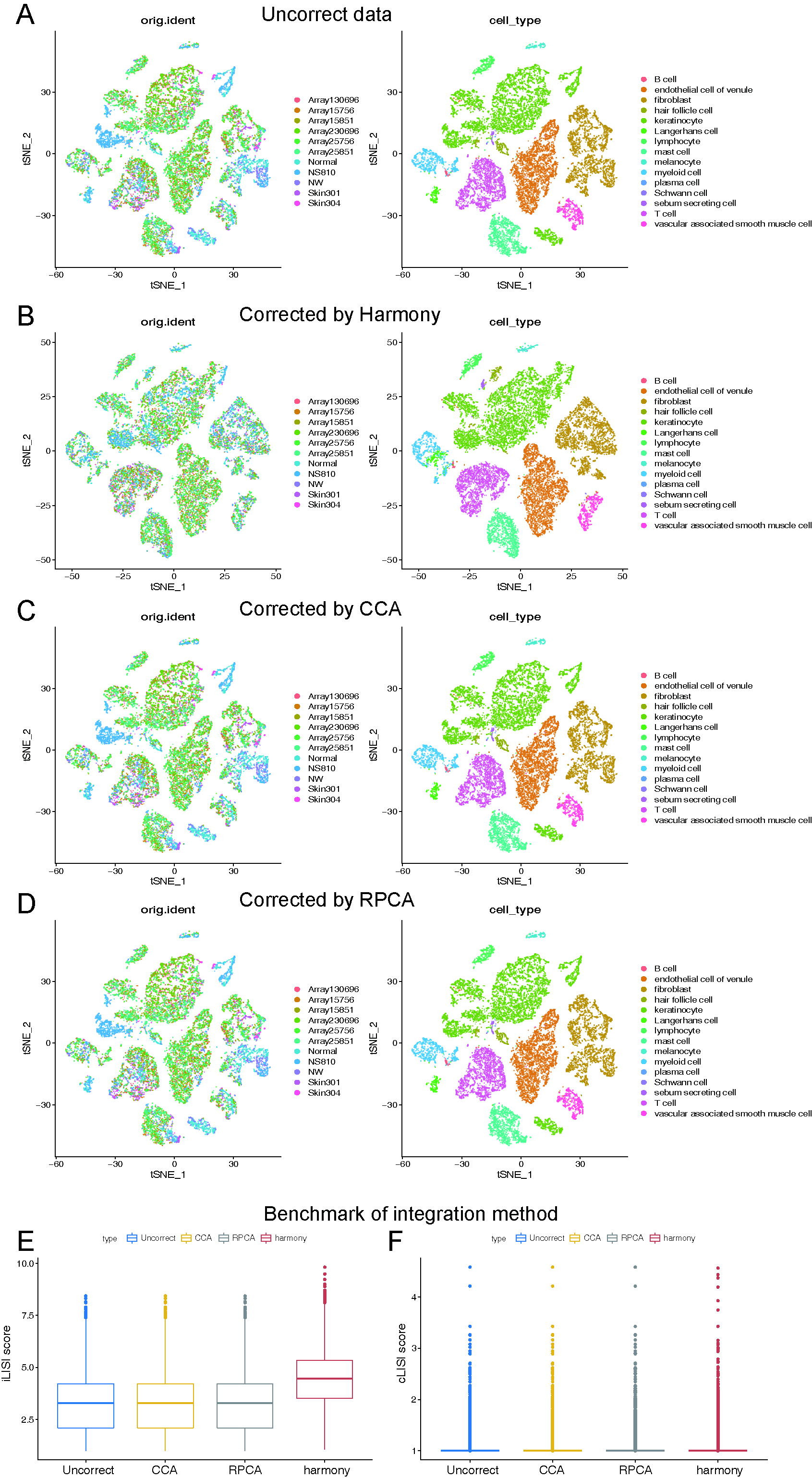

Supplement: Supplementary file 12 [file Image5.TIF]
